# Integrin-α_V_-mediated activation of TGF-β regulates anti-tumour CD8 T cell immunity and response to PD-1 blockade

**DOI:** 10.1038/s41467-021-25322-y

**Published:** 2021-09-01

**Authors:** Ines Malenica, Julien Adam, Stéphanie Corgnac, Laura Mezquita, Edouard Auclin, Isabelle Damei, Laetitia Grynszpan, Gwendoline Gros, Vincent de Montpréville, David Planchard, Nathalie Théret, Benjamin Besse, Fathia Mami-Chouaib

**Affiliations:** 1 INSERM UMR 1186, Integrative Tumour Immunology and Immunotherapy, Gustave Roussy, Fac. de Médecine—Univ. Paris-Sud, Université Paris-Saclay, Villejuif, France; 2 Department of Cancer Medicine, Gustave Roussy Cancer Campus, Institut d’Oncologie Thoracique, Gustave Roussy, Université Paris-Saclay, Villejuif, France; 3 Medical and Thoracic Oncology Department, Hôpital Européen Georges Pompidou, AP-HP, Paris, France; 4 Hôpital Marie-Lannelongue, Service d’Anatomie Pathologique, Le-Plessis-Robinson, France; 5 Univ Rennes, Inserm, EHESP, Irset-UMR-S1085, Rennes, France; 6 Present Address: Laboratory of Translational Genomics and Targeted Therapeutics in Solid Tumours, August Pi i Sunyer Biomedical Research Institute (IDIBAPS), Barcelona, Spain; 7 Present Address: Medical Oncology Department, Hospital Clínic, Barcelona, Spain

**Keywords:** Non-small-cell lung cancer, Tumour immunology, Translational immunology, Translational research

## Abstract

TGF-β is secreted in the tumour microenvironment in a latent, inactive form bound to latency associated protein and activated by the integrin α_V_ subunit. The activation of latent TGF-β by cancer-cell-expressed α_V_ re-shapes the tumour microenvironment, and this could affect patient responses to PD-1-targeting therapy. Here we show, using multiplex immunofluorescence staining in cohorts of anti-PD-1 and anti-PD-L1-treated lung cancer patients, that decreased expression of cancer cell α_V_ is associated with improved immunotherapy-related, progression-free survival, as well as with an increased density of CD8^+^CD103^+^ tumour-infiltrating lymphocytes. Mechanistically, tumour α_V_ regulates CD8 T cell recruitment, induces CD103 expression on activated CD8^+^ T cells and promotes their differentiation to granzyme B-producing CD103^+^CD69^+^ resident memory T cells via autocrine TGF-β signalling. Thus, our work provides the underlying principle of targeting cancer cell α_V_ for more efficient PD-1 checkpoint blockade therapy.

## Abstract (web summary)

Response to PD-1 checkpoint blockade is unpredictable in lung cancer patients. Here authors show in human lung and mouse tumour models that low or absent α_V_ integrin expression leads to better tumour growth control by anti-PD-1 via reduced TGF-β activation and hence increased infiltration of anti-tumour CD8^+^ T cells.

## Introduction

Immunotherapy targeting the T cell inhibitory receptor programmed death-1 (PD-1) and its ligand PD-L1 holds promise in lung cancer treatment^1^. However, given the limited therapeutic benefit of anti-PD-(L)1 antibodies as single agents, it is crucial to identify the immune mechanisms involved in tumour resistance to immune checkpoint blockade (ICB) and develop more effective combinatorial approaches. Resistance to ICB has been associated with defects in genes relating to antigen presentation by major histocompatibility complex class I (MHC-I) molecules to CD8^+^ T lymphocytes^2–4^, mutations in the Janus kinase (JAK)1/JAK2 and interferon (IFN) signalling pathways^2,5,6^, and clonal deletion of tumour-specific T cells^7^. Accumulating evidence indicates that tumour regression following PD-1 blockade requires pre-existing CD8^+^ T lymphocytes that are negatively regulated by PD-1-mediated resistance^8^. Consequently, tumours that are weakly infiltrated by CD8^+^ T cells are unlikely to respond to such therapeutic interventions. The quality of CD8^+^ tumour-infiltrating lymphocytes (TIL), especially their reactivity toward the cognate target, is also directly associated with the efficacy of anti-PD-1. In this regard, expression of PD-1 on CD8^+^ TIL appeared to define clonally expanded tumour neoantigen-specific T cells detected in cancer patients^9,10^. More recently, expression of CD103 (α_E_(CD103)β_7_) integrin, together with CD39 and CD137 (4-1BB), has been reported to identify truly tumour-reactive CD8^+^ T cells in human solid tumours^11,12^.

CD103 delineates a subtype of CD8^+^ resident memory T (T_RM_) cells, which stably reside in many human solid tumours where they likely orchestrate a local immune response to cancer cells^13^. Human non-small-cell lung cancer (NSCLC) CD103^+^CD8^+^ T_RM_ cells frequently express the activation marker CD69 and a panel of T cell inhibitory receptors, including PD-1^14^. They are enriched with tumour-reactive T lymphocytes able to kill autologous tumour cells upon blockade of PD-1 with neutralizing antibodies^15^. This CD103^+^CD8^+^ T_RM_ subset emerges as a predictive marker of survival in several cancers, including NSCLC^15–18^. CD103^+^CD8^+^ T_RM_ expands during anti-PD-1 immunotherapy, and their accumulation in tumours is associated with the improved outcome of anti-PD-(L)1-treated patients^14,18,19^.

It is now widely recognized that induction of CD103 on activated CD8^+^ T lymphocytes and persistence of CD103^+^CD8^+^ T_RM_ in epithelial tissues requires transforming growth factor-beta (TGF-β). TGF-β is important for tissue remodelling and repair at sites of inflammation^20^, but is also an immunosuppressive mediator used by malignant cells to escape from the immune system^21,22^. This cytokine is secreted in the tumour microenvironment (TME) in its inactive (latent) form bound to latency-associated protein (LAP), and is activated by metalloproteinase*s* (MMP)^23,24^ and RGD-binding integrins, such as α_V_β_6_ and α_V_β_8_^25,26^. It has been reported that α_V_β_8_-expressing tumours evade host immunity by activating latent TGF-β on adjacent immune cells^27^. In contrast, activation of TGF-β by α_V_β_8_ integrin on tumour-infiltrating dendritic cells (DC) induced CD103 expression on CD8^+^ T cells resulting in inhibition of cancer progression^28^. Thus, the role of α_V_ integrins in shaping the tumour ecosystem and regulating the anti-tumour immune response is controversial and needs to be better understood.

Here, we show that increased tumour α_V_ expression is associating with worse immunotherapy-related progression-free survival (PFS) in anti-PD-(L)1-treated NSCLC patients, which correlates with the decreased density of CD103^+^CD8^+^ TIL. In vivo therapeutic blockade of PD-1 in a mouse model greatly improves growth control of α_V_-knockout tumours via a mechanism involving increased tumour infiltration by activated tumour-specific CD103^+^CD8^+^ T cells. Thus, targeting tumour α_V_ integrin to prevent endogenous TGF-β maturation is a promising approach for more effective ICB.

## Results

### Tumour α_V_ expression levels influence response to anti-PD-(L)1

**Fig. 1:**
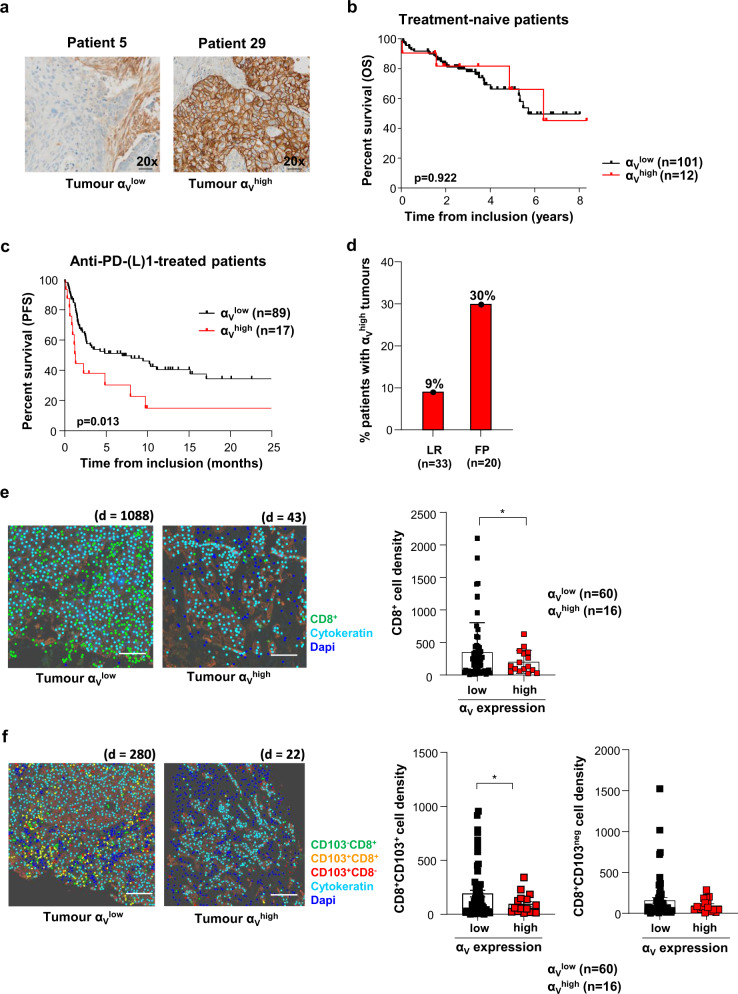
Decreased tumour α_V_ expression correlates with improved anti-PD-(L)1-treated NSCLC patient survival. **a** Representative IHC images of tumour samples from patients with low and high α_V_ expression in tumour cells. Objective: 20×. **b** Kaplan−Meier curve of OS for stage I treatment-naïve lung cancer patients according to the α_V_ expression by IHC analysis of FFPE tumours. **c** Kaplan−Meier curve of PFS of PD-1 blockade-treated patients with tumours harbouring low and high expression of α_V_ integrin. **d** Percentages of anti-PD-(L)1-treated patients displaying α_V_^high^ tumours among long-responders (LR: PFS > 6 months and OS > 12 months) or fast progressors (FP: defined by “early death” occurring within 12 weeks of treatment initiation). **e** Representative digital mark-up image of fluorescent IHC of CD8 (green), cytokeratin (turquoise), and dapi (blue) staining in α_V_^low^ and α_V_^high^ tumour sections. *d* = CD8^+^ cell density. Left, the density of CD8^+^ TIL in α_V_^low^ and α_V_^high^ tumours. The numbers of tumours in each group are indicated (*p = 0.046). Scale bar, 2 cm. **f** Representative digital mark-up image of CD8^+^CD103^neg^ (green), CD8^+^CD103^+^ (orange), CD8^-^CD103^+^ (red), cytokeratin (turquoise) and dapi (blue) staining in α_V_^low^ and α_V_^high^ tumour sections. *d* = CD8^+^CD103^+^ cell density. Left, the density of CD8^+^CD103^+^ (*p = 0.016) and CD8^+^CD103^neg^ (*p* = 0.120) cells in tumour regions of α_V_^low^ and α_V_^high^ tumours. Scale bar, 2 cm. Each symbol represents an individual cell type from tumour samples; horizontal lines correspond to mean ± standard error of the mean (SEM) (**e**, **f**). Data were calculated with the log-rank test (**b**, **c**) and Welch’s two-sided *t*-test (**e**, **f**). Source data are provided as a Source Data file.

To investigate the impact of tumour α_V_ expression on survival in patients with lung cancer, we used a retrospective cohort of 113 patients with treatment-naïve early-stage NSCLC^15^. Tumour sections from formalin-fixed, paraffin-embedded samples were stained with anti-α_V_ monoclonal antibodies (mAb) and evaluated by immunohistochemistry (IHC) for the expression of the integrin in epithelial tumour regions. Variability in tumour α_V_ expression was seen, with 11% of tumours displaying a α_V_^high^ profile and 89% displaying a α_V_^low^ profile, among which 36% were negative for α_V_ expression (α_V_^neg^) (Fig. 1a). We did not find any significant difference in overall survival (OS) of patients bearing α_V_^low^ and α_V_^high^ tumours (Fig. 1b), with a hazard ratio (HR) = 1.05, 95% confidence interval (CI) 0.38−2.97, and median OS of 68 months (95% CI 62.9−not reached). Similar non-significant results were obtained with public TCGA datasets from therapy-naïve stage I lung cancers (Supplementary Fig. 1a). These data indicate that tumour α_V_ expression does not influence treatment-naïve patient survival.

We then examined the consequence of tumour α_V_ levels on survival in patients treated with ICB. We established a retrospective cohort (cohort 1) of 106 patients with advanced NSCLC treated with a single-agent anti-PD-(L)1 as a second-line treatment (Supplementary Table 1)^14^. Association of α_V_ expression in epithelial tumour regions with progression-free survival (PFS) after the first immunotherapy administration was assessed. Notably, chemotherapy and radiation therapy prior to immunotherapy had no significant impact on tumour α_V_ levels (Supplementary Table 1). Patients with α_V_^low^ tumours (including 18% α_V_^neg^) had increased PFS with a HR = 0.47 (95% CI 0.17−0.81, *p* = 0.01) and a median PFS of 8.2 months (Fig. 1c), compared to patients with α_V_^high^ tumours (16% of patients), who displayed strongly decreased PFS, with a median PFS of 1.5 months. Association between α_V_^low^ tumours and better PFS of anti-PD-(L)1-treated patients was then investigated in a second cohort (cohort 2) of 51 NSCLC (Supplementary Table 2 and Supplementary Fig. 1b) alone and pooled with cohort 1 (Supplementary Fig. 1c) because of the low number (*n* = 9) of α_V_^high^ tumours in cohort 2. Results showed a trend toward statistical significance for the association between α_V_^low^ tumours and better PFS in anti-PD-(L)1-treated patients (Supplementary Fig. 1c). In multivariable analysis performed on the pooled cohorts (*n* = 157), there was a trend toward a worse PFS for patients with α_V_^high^ tumours (HR = 1.60, 95% CI 0.98−2.62, *p* = 0.06) (Supplementary Table 3). Notably, tumours with α_V_^high^ expression levels in the cohort 1 were predominant in fast-progressor patients, defined by “early death” occurring during the first 12 weeks after initiating ICB than in long-responder patients, defined by a PFS > 6 months and OS > 12 months. Indeed, 30% of fast-progressors displayed α_V_^high^ tumours versus 9% of long-responders (Fig. 1d). More importantly, quantitative multiplex fluorescent IHC staining using anti-CD8 mAb showed that α_V_^low^ tumours were more strongly infiltrated with CD8^+^ lymphocytes than α_V_^high^ tumours, with a CD8 cell density ranging from 8 to 2103 cells/mm^2^ and a median of 352 cells/mm^2^ in α_V_^low^ tumours; and from 25 to 436 cells/mm^2^ and a median of 176 cells/mm^2^ in α_V_^high^ tumours (*p* = 0.046; Fig. 1e). Similar results were obtained when we pooled cohort 1 and cohort 2 (Supplementary Fig. 1d). Furthermore, multiplex IHC staining with anti-CD8 and anti-CD103 mAb showed that the density of CD8^+^CD103^+^ cells was enhanced in tumours with α_V_^low^ levels compared to tumours with α_V_^high^ (Fig. 1f and Supplementary Fig. 1d). A difference in CD8^+^CD103^neg^ cell infiltration was observed when we pooled both patient cohorts (*p* = 0.002; Supplementary Fig. 1d). These results support the observation that α_V_ integrin dictates patient response to ICB by regulating tumour infiltration by CD8^+^ lymphocytes. They suggest that by activating TGF-β, α_V_ participates in CD8 T cell exclusion from the TME and differentiation of CD103^+^ T_RM_ cells.

### Integrin α_V_ on NSCLC cells activates TGF-β

**Fig. 2:**
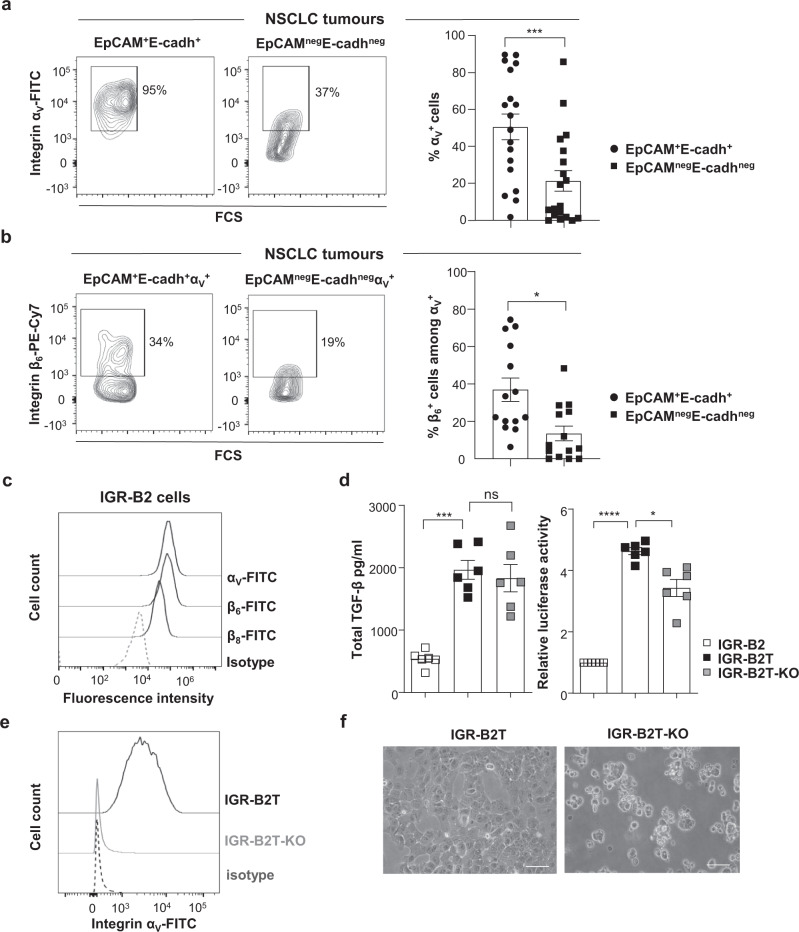
Human NSCLC tumour cells express α_V_ integrin, which activates autocrine TGF-β. **a** Representative flow cytometry plots (bi-exponential scale) of α_V_ expression in EpCAM^+^E-cadherin^+^ and EpCAM^neg^E-cadherin^neg^ cells from a lung tumour. Right, percentage of α_V_ expression in EpCAM^+^E-cadherin^+^ and EpCAM^neg^E-cadherin^neg^ cells (*n* = 18, ***p = 0.0002). **b** Representative flow cytometry plots of β_6_ subunit expression in EpCAM^+^E-cadherin^+^α_V_^+^ and EpCAM^neg^E-cadherin^neg^α_V_^+^ cells from a tumour sample. Right, expression of β_6_ integrin in EpCAM^+^E-cadherin^+^α_V_^+^ and EpCAM^neg^E-cadherin^neg^α_V_^+^ cells (*n* = 16), *p = 0.013. **c** Surface expression of α_V_, β_6_, and β_8_ subunits in the IGR-B2 cell line. **d** Concentration of total TGF-β in CM from IGR-B2, IGR-B2T, and IGR-B2T-KO cells measured by ELISA (***p = 0.0004). Results are presented as mean ± SEM of six independent experiments. Right, relative luciferase activity in the Mu.1LV cell line transfected with (CAGA)9-Lux reporter plasmid and treated with CM from IGR-B2, IGR-B2T, and IGR-B2T-KO cells, normalized to luciferase activity in Mu.1LV cell treated with CM from IGR-B2. Results are presented as mean ± SEM of six independent experiments (*p = 0.011, ****p < 0.0001). **e** Expression of α_V_ integrin on IGR-B2T and IGR-B2T-KO cells. An isotype control was included. **f** Representative photos of the morphology of IGR-B2T and IGR-B2T-KO cells by phase-contrast light microscope from one experiment out of five. Objective: 20×. Each symbol represents the individual cell type from tumour samples (**a**, **b**); horizontal lines correspond to mean ± SEM (**a**, **b**, **d**). Data were calculated with paired Student *t*-tests (**a**, **b**) and one-way ANOVA with Tukey’s correction (**d**). ns: non-significant. Source data are provided as a Source Data file.

Next, we investigated the expression of α_V_ in NSCLC tumours ex vivo. Because the α_V_ subunit pairs with β_6_ and β_8_ subunits to activate TGF-β^26^, we first conducted quantitative (q)RT-PCR to evaluate the expression of *ITGAV*, *ITGB6,* and *ITGB8* mRNA in 15 freshly resected primary NSCLC. Log scale analyses showed that *ITGAV* and *ITGB8* transcripts were more strongly expressed in four out of 15 tumour samples compared to autologous proximal healthy lung tissues. In contrast, the *ITGB6* transcript was much less frequently expressed (Supplementary Fig. 2a). qRT-PCR conducted to evaluate the expression of genes encoding additional β subunits that may pair with α_V_ integrin showed that *ITGB1* and *ITGB5* mRNA were equally expressed in tumours than in adjacent healthy lungs (*n* = 15) and that *ITGB3* was more strongly expressed in two tumours than in healthy lungs (Supplementary Fig. 2b). We then assessed α_V_ protein expression in 18 additional freshly resected tumours using specific mAb, combined with anti-EpCAM and anti-E-cadherin to delineate integrin expression on epithelial cancer cells (Supplementary Fig. 2c). Multi-parametric immunofluorescence showed variable α_V_ levels with a mean of 51 ± 7% of EpCAM^+^E-cadherin^+^ tumour cells that expressed the integrin, while only 21 ± 6% of non-epithelial EpCAM^neg^E-cadherin^neg^ cells were α_V_^+^ (Fig. 2a). Moreover, 37 ± 6% of EpCAM^+^E-cadherin^+^ tumour cells expressed α_V_ in association with β_6_, while only 14% ± 4% of non-epithelial α_V_^+^EpCAM^neg^E-cadherin^neg^ cells were β_6_^+^, suggesting that α_V_ pairs with β_8_ or β_3_ in α_V_^+^β_6_^neg^ cells (Fig. 2b and Supplementary Fig. 2c). It should be noted that CD4^+^ and CD8^+^ T cells from NSCLC TIL and healthy donor (HD) peripheral blood mononuclear cells (PBMC) only rarely expressed the integrin (Supplementary Fig. 2c and d). To select a NSCLC cell line that expresses α_V_ for further studies, we tested a panel of 13 cell lines by FACS. While α_V_ was expressed in nine cell lines, the β_6_ and β_8_ subunits were much less frequently expressed (Supplementary Table 4). Among these cell lines, we retained the IGR-B2 because it expressed the three subunits that likely form α_V_β_6_ and α_V_β_8_ heterodimers (Fig. 2c). In addition, qRT-PCR showed that IGR-B2 did not express *ITGB1* and *ITGB5* genes, which were over-expressed in five and four cell lines as compared to 16HBE human bronchial epithelial cells (Supplementary Table 5), but over-expressed, together with seven other cell lines, *ITGB3*, suggesting that α_V_ may also pair with β_3_ in these cells. Thus, the role of other α_V_ integrins in this human tumour model cannot be ruled out.

We then assessed the capacity of α_V_ integrins on IGR-B2 to activate LAP-TGF-β. Since IGR-B2 does not produce high levels of TGF-β, although *TGFB1* mRNA was detected by qRT-PCR (Supplementary Table 5), we first transfected the cell line with a human LAP-TGF-β-encoding plasmid and selected a clone (IGR-B2T) producing strong levels of TGF-β (Fig. 2d). Using the CRISPR-Cas9 system, we also generated from IGR-B2T a cell clone knockout (KO) for the α_V_ subunit (IGR-B2T-KO) to suppress all α_V_ integrins (Fig. 2e). Notably, deletion of the *ITGAV* gene in IGR-B2T-KO cells resulted in dramatic morphology changes with loss of the adhesive capacity and formation of spheroids (Fig. 2f). Nevertheless, these changes did not affect IGR-B2T-KO susceptibility to autologous CTL clone-mediated killing, excluding alteration in target cell recognition (Supplementary Fig. 2e). We then conducted experiments to determine the capacity of α_V_ integrins on IGR-B2T cells to activate LAP-TGF-β. Results showed that while IGR-B2T and IGR-B2T-KO produced equal levels of LAP-TGF-β, IGR-B2T-KO cells produced lower quantities of active TGF-β as measured by luciferase activity (Fig. 2d). However, inhibition of TGF-β activation in IGR-B2T-KO was incomplete, suggesting that additional mechanisms may activate the cytokine.

**Fig. 3:**
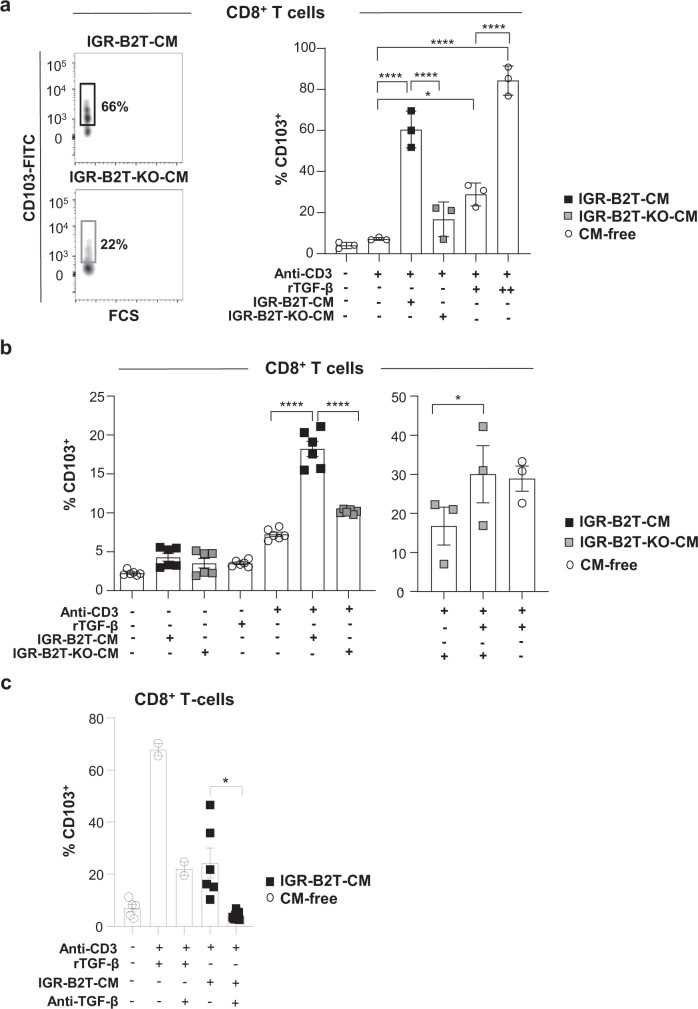
CM of IGR-B2T cells promotes CD103 expression in activated human CD8^+^ T cells. **a** Representative flow cytometry plots (bi-exponential scale) of the expression of CD103 in CD8^+^ T cells from HD PBMC stimulated for three days with plastic-coated anti-CD3 mAb in the presence of CM from IGR-B2T or IGR-B2T-KO cells. Right, percentages of CD103^+^ cells among CD8^+^ T cells of non-stimulated and stimulated PBMC (*n* = 3). Recombinant (r)TGF-β at 1 ng/ml (+) or 5 ng/ml (++) was used as a positive control (*p = 0.011, ****p < 0.0001). **b** Percentages of CD103^+^ cells among CD8^+^ T lymphocytes from HD PBMC unstimulated or stimulated for three days with anti-CD3 alone, rTGF-β alone, CM alone, and a combination of anti-CD3 plus CM from IGR-B2T or IGR-B2T-KO cells (*n* = 6). Right, rescue experiments. Percentages of CD103^+^ lymphocytes among CD8^+^ T cells stimulated with a combination of anti-CD3 plus CM from IGR-B2T-KO cells, CM from IGR-B2T-KO plus low dose (1 ng/ml) of rTGF-β, and anti-CD3 plus rTGF-β, included as a control (*n* = 3, *p = 0.048, ****p < 0.0001). **c** Inhibition of CD103 induction with anti-TGF-β blocking mAb. Percentages of CD103^+^ lymphocytes among CD8^+^ T cells stimulated with CM from IGR-B2T plus anti-CD3 in the absence and presence of anti-TGF-β mAb (*n* = 6, *p = 0.022). A combination of anti-CD3 and rTGF-β alone and in presence of neutralizing anti-TGF-β was included as a control. Each symbol represents an individual cell type. Horizontal lines correspond to mean ± SEM. Data were calculated with one-way ANOVA with Tukey’s correction (**a**, **b**) and paired Student *t*-test (**c**). Source data are provided as a Source Data file.

Next, we asked whether tumour-derived active TGF-β participates in the formation of T_RM_ cells such as by inducing CD103^29,30^. We stimulated HD PBMC with plastic-coated anti-CD3 mAb in the presence of conditioned medium (CM) from IGR-B2T or IGR-B2T-KO cells, and evaluated CD103 expression on CD8^+^ T cells. Activation of T lymphocytes with anti-CD3 plus CM from IGR-B2T resulted in induction of CD103 on up to 70% of CD8^+^ T cells (Fig. 3a and Supplementary Fig. 3). In contrast, stimulation of PBMC with anti-CD3 plus CM from IGR-B2T-KO triggered CD103 expression on only up to 22% of CD8^+^ T lymphocytes. Stimulation with anti-CD3 alone or CM alone did not induce CD103 expression, and a combination of anti-CD3 plus recombinant (r)TGF-β, used as a positive control, induced expression of the integrin in a dose-dependent manner (Fig. 3b). Moreover, the supply of rTGF-β to CM from IGR-B2T-KO resulted in increased expression of CD103 (Fig. 3b). In contrast, the addition of anti-TGF-β neutralizing mAb to CM from IGR-B2T inhibited CD103 induction (Fig. 3c). These data indicate that α_V_ integrins on tumour cells activate TGF-β and thereby regulate CD103 expression on activated CD8^+^ T cells.

### Tumour α_V_ shapes the TME by activating TGF-β

The above data suggest that by activating TGF-β, tumour α_V_ determines the nature of CD8^+^ T cell infiltrate and therefore anti-tumour T cell immunity and response to immunotherapy. To test this hypothesis, we developed an in vivo model of C57BL/6 mice engrafted with B16F10 melanoma cells transfected with the CD103 ligand E-cadherin (B16F10E) to mimic epithelial tumours. We selected B16F10 because it expressed α_V_ and β_8_ subunits, produced constitutively LAP-TGF-β (Supplementary Fig. 4a−c) and is known to respond to ICB^31^. Notably, B16F10E does not express β_1_, β_3_, β_5,_ and β_6_ subunits tested by qRT-PCR and/or FACS (Supplementary Fig. 4a). Using the CRISPR-Cas9 system, we generated a cell clone deficient for α_V_ (B16F10E-KO) which displayed similar expression levels of E-cadherin as the parental clone (Supplementary Fig. 4d). As for human IGR-B2-KO, we observed dramatic morphological changes in B16F10E-KO cells compared to B16F10E, with loss of plastic adhesive capacity (Supplementary Fig. 4b). Moreover, luciferase activity assay showed that B16F10E-KO produced lower levels of active TGF-β than B16F10E even though both tumour cell clones secreted similar levels of the cytokine latent form (Supplementary Fig. 4c). Activation of LAP-TGF-β was associated with the interaction of α_V_ integrin with LAP-TGF-β in B16F10E, but not in B16F10E-KO negative control, as examined by proximity ligation assay (PLA) and confocal microscopy (Supplementary Fig. 4e). In addition, CM from B16F10E, and to a much lesser extent from B16F10E-KO, induced expression of CD103 on T cell-receptor (TCR)-engaged CD8^+^ T cells from HD PBMC (Supplementary Fig. 4f). Like for IGR-B2T-KO, inhibition of TGF-β activation in B16F10E-KO cells was partial, supporting the hypothesis that additional mechanisms, such as metalloproteases (MMP)^23,24^, are involved in the maturation of LAP-TGF-β. Consistently, B16F10E expressed MMP14, previously shown to release latent TGF-β from cell-extracellular matrix^23^ (Supplementary Fig. 4g). Moreover, stimulation of HD PBMC with anti-CD3 plus CM from B16F10E treated with the MMP inhibitor GM6001 resulted in decreased expression of CD103 on CD8^+^ T cells as compared to CM from untreated cells (Supplementary Fig. 4h).

**Fig. 4:**
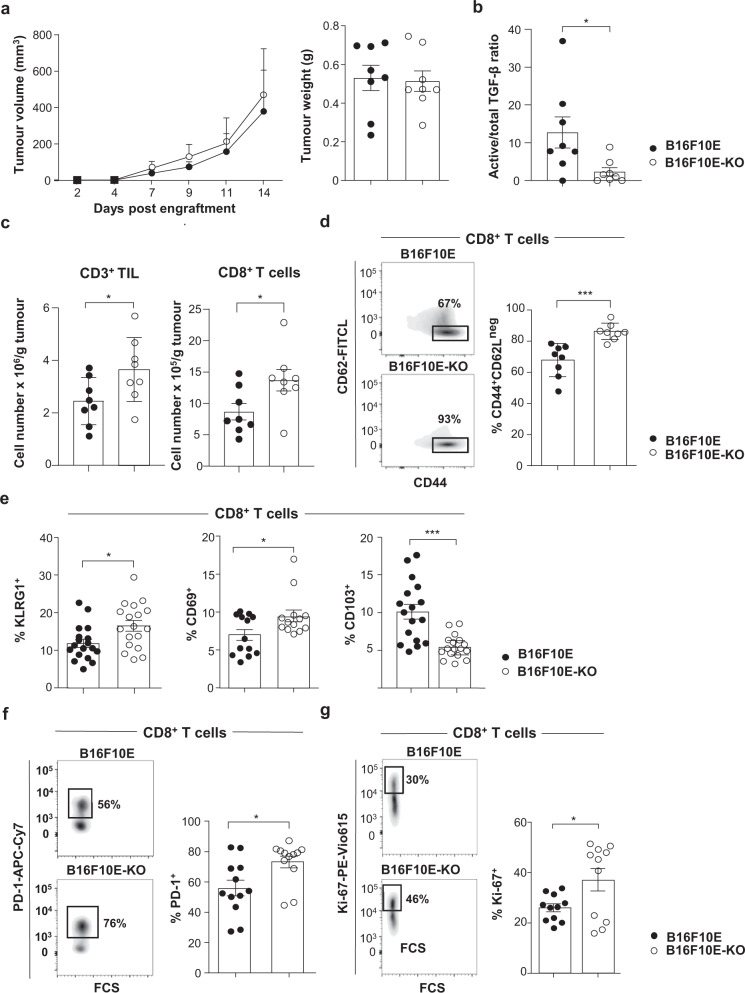
α_V_^high^ tumours promote TIL exclusion and decreased T cell proliferation and activation. **a** Left, B16F10E and B16F10E-KO tumour growth kinetics (*p* = 0.800). Right, tumour weights of B16F10E and B16F10E-KO tumours recovered at day 14 after engraftment (*p* = 0.846). Tumour volumes and tumour weights are given as means ± SEM of eight mice/group. Data represent one independent experiment out of three. **b** The ratio of active versus total TGF-β from B16F10E and B16F10E-KO tumours (*n* = 8) was measured ex vivo on day 8 by ELISA (*p = 0.038). **c** Absolute cell counts of CD3^+^ (*p = 0.040) and CD8^+^ T cells (*p = 0.034) infiltrating B16F10E and B16F10E-KO tumours (*n* = 8). Data are from one independent experiment out of three. **d** Representative flow cytometry profiles (bi-exponential scale) of the expression of CD62L and CD44 in CD8^+^ T cells from B16F10E and B16F10E-KO tumours (*n* = 8). Right, percentage of CD44^+^CD62L^neg^ cells among CD8^+^ T cells in B16F10E and B16F10E-KO (***p = 0.0006). **e** KLRG1 (*n* = 18, *p = 0.014), CD69 (*n* = 13, *p = 0.025) and CD103 (*n* = 17, ***p = 0.0003) expression in CD8^+^ T cells infiltrating B16F10E and B16F10E-KO tumours. Data are from two independent experiments out of three. **f** Representative flow cytometry profiles of PD-1 expression on CD8^+^ TIL from B16F10E and B16F10E-KO. Right, expression of PD-1 on CD8^+^ T cells infiltrating B16F10E and B16F10E-KO tumours (*n* = 12, *p = 0.016). **g** Representative profiles of Ki-67 expression in CD8^+^ T cells from B16F10E and B16F10E-KO. Right, expression of Ki-67 in CD8^+^ T cells from B16F10E and B16F10E-KO tumours (*n* = 11, *p = 0.028). Data are from two independent experiments out of three. Each symbol represents an individual tumour (**a**−**g**.). Horizontal lines correspond to mean ± SEM (**a**, right−**g**.). Data were calculated with unpaired Student *t*-tests (**a**, right−**g**.) and two-way ANOVA (**a**, left). Source data are provided as a Source Data file.

To examine the impact of α_V_ integrins on anti-tumour T cell response in vivo, we engrafted B16F10E and B16F10E-KO cells in C57BL/6 mice and followed tumour development. We first verified that both tumour cell clones proliferated equally in vitro, using the CFSE assay, and in vivo, after engraftment into immune-deficient nude mice (Supplementary Fig. 5a, b). Implanting B16F10E and B16F10E-KO into immune-competent C57BL/6 mice resulted in similar tumour growth kinetics and tumour weights (Fig. 4a). In these series of experiments, we checked that B16F10E tumours produced higher levels of active TGF-β than B16F10E-KO ex vivo (Fig. 4b). Notably, CD8^+^ and CD4^+^ TIL from both B16F10E and B16F10E-KO expressed the α_V_ integrin, which may participate in TGF-β activation (Supplementary Figs. 5c and 6a). More importantly, B16F10E-KO tumours were more strongly infiltrated with CD3^+^ and CD8^+^ T cells than B16F10E (Fig. 4c). Moreover, CD8^+^ T cells from B16F10E-KO were more enriched with CD44^+^CD62L^neg^ effector cells than those from B16F10E (Fig. 4d). In contrast, both tumours were equally infiltrated with CD4^+^ T cells, which included similar percentages of CD44^+^CD62L^neg^ cells (Supplementary Fig. 6b). B16F10E and B16F10E-KO tumours were also equally infiltrated with CD103^+^CD45^+^CD3^-^MHC-II^+^CD11c^+^ DC (Supplementary Fig. 6c). Remarkably, CD8^+^ T cells from B16F10E-KO were more enriched with KLRG1^+^ effectors and activated CD69^+^ cells, and less enriched with CD103^+^ cells than B16F10E (Fig. 4e), which correlated with a decreased production of active TGF-β by the former tumours. Consistent with this, transcription factors Smad2/3 were less frequently phosphorylated in CD8^+^ T lymphocytes from B16F10E-KO than B16F10E tumours (Supplementary Fig. 6d). CD8^+^ T cells from B16F10E-KO were also more highly enriched with activated PD-1^+^ and proliferative Ki-67^+^ cells than B16F10E emphasizing a higher activation state (Fig. 4f, g). These results indicate that knockout of tumour α_V_ results in increased recruitment and activation of CD8^+^ T cells, most likely linked to modulation of TGF-β maturation.

### Knockout of α_V_ improves CD8 T cell immunity and response to anti-PD-1

**Fig. 5:**
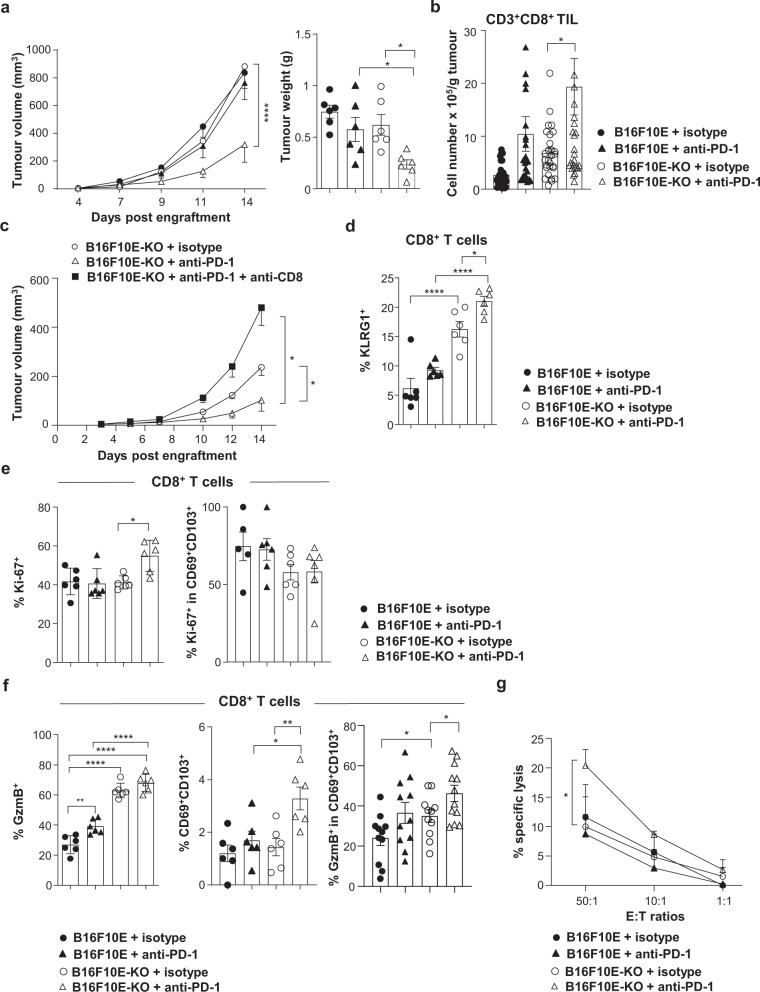
Anti-PD-1 improves tumour growth control of B16F10E-KO. **a** Growth of B16F10E and B16F10E-KO tumours (****p < 0.0001). Right, weights of tumours recovered at day 14, B16F10E+anti-PD-1 vs. B16F10E-KO+anti-PD-1 (*p = 0.023), B16F10E-KO+isotype vs. B16F10E-KO+ anti-PD-1 (*p = 0.049). Tumour volumes and weights are given as means± SEM (*n* = 6). Data represent one independent experiment out of five. **b** Absolute number of CD8^+^ T cells from B16F10E (*n* = 16) and B16F10E-KO (*n* = 18) tumours treated with anti-PD-1 or isotype control (*n* = 17, B16F10E; *n* = 19, B16F10E-KO). Data are from five independent experiments (*p = 0.037). **c** Growth of B16F10E-KO tumours (*n* = 6). Tumour volumes are given as means ± SEM. Data are from one independent experiment out of two. B16F10E-KO+iso vs. B16F10E-KO+anti-PD-1 (*p = 0.043); B16F10E-KO+anti-PD-1 vs. B16F10E-KO+anti-CD8 (*p = 0.020). **d** Percentages of KLRG1^+^ cells in CD8^+^ T cells from tumours (*n* = 6) treated with anti-PD-1 or isotype control (*p = 0.043, ****p < 0.0001). Data are from one independent experiment out of three. **e** Percentages of Ki-67^+^ T cells among CD8^+^ (*p = 0.012) and CD69^+^CD103^+^CD8^+^ TIL from B16F10E-KO (*n* = 6) and B16F10E tumours treated with anti-PD-1 (*n* = 6) or isotype control (*n* = 5). Data are from one independent experiment out of three. **f** Percentages of granzyme B (GzmB)^+^ (left, **p = 0.0043, ****p < 0.0001) and CD69^+^CD103^+^ (middle, *p = 0.025, **p = 0.008) in CD8^+^ T cells from tumours (*n* = 6) treated with anti-PD-1 or isotype control. One independent experiment out of three is shown. Right, percentages of granzyme B^+^ cells in CD69^+^CD103^+^CD8^+^ T cells from B16F10E (*n* = 11) and B16F10E-KO (*n* = 12) treated with anti-PD-1 or isotype control (*n* = 11), B16F10E+iso vs. B16F10E-KO+isotype (*p = 0.046); B16F10E-KO+isotype vs. B16F10E-KO+anti-PD-1 (*p = 0.040). Two independent experiments out of three are included. **g**. Cytotoxic activity of CD8^+^ TIL isolated from B16F10E and B16F10E-KO tumours treated with anti-PD-1 or isotype control (*p = 0.037). Indicated are the effector-to-target (E:T) ratios. Data are means of three independent experiments. Each symbol represents an individual tumour; horizontal lines correspond to mean ± SEM (**a** right, **b**, **d**−**f**.). Data were calculated with one-way ANOVA with Tukey’s correction (**a** right, **d**, **e**, **f** left, middle), unpaired *t*-test (**b**, **f** right, **g**.), two-way ANOVA for tumour growth (**a**, **c**). Source data are provided as a Source Data file.

The above-described human studies showed that tumour α_V_ expression levels influence the outcome of anti-PD-1-treated NSCLC patients via a mechanism dependent on increased density of CD8^+^ TIL. To evaluate the impact of tumour α_V_ expression on response to ICB in vivo, we engrafted C57BL/6 mice with B16F10E and B16F10E-KO cells, treated them with anti-PD-1, and monitored tumour progression. Results indicated that intraperitoneal (i.p.) administration of neutralizing anti-PD-1 mAb greatly improved control of B16F10E-KO tumour growth, but not B16F10E growth (Fig. 5a). In contrast, isotype control-treated mice displayed similar tumour progression kinetics and tumour weight. B16F10E-KO growth control in anti-PD-1-treated mice was associated with an increase in tumour infiltration by CD8^+^ T cells compared to isotype control-treated mice (Fig. 5b), and this benefit was abolished when mice received anti-CD8 blocking mAb (Fig. 5c).

Next, we examined the consequence of tumour α_V_-knockout combined with PD-1 blockade on the quality and functionality of CD8^+^ TIL. Multi-parametric immunofluorescence analyses indicated that CD8^+^ TIL from anti-PD-1-treated B16F10E-KO tumours was more enriched with terminally differentiated KLRG1^+^ effector T cells than those from isotype-treated B16F10E-KO and anti-PD-1-treated B16F10E (Fig. 5d). Notably, CD8^+^ TIL from anti-PD-1-treated B16F10E-KO was only marginally less enriched with CD103^+^ T cells than those from isotype-treated B16F10E-KO and anti-PD-1-treated B16F10E tumours (Supplementary Fig. 7a). In contrast, much lower percentages of CD103^+^ cells among FoxP3^+^CD4^+^ Treg were observed in B16F10E-KO tumours than in B16F10E (Supplementary Fig. 7b). Moreover, CD8^+^ T lymphocytes from anti-PD-1-treated B16F10E-KO, but not from B16F10E, displayed increased percentages of Ki-67^+^ cells as compared to isotype-treated B16F10E-KO suggesting that PD-1 blockade promoted CD8^+^ T cell expansion in the former tumours associated with decreased TGF-β activation (Fig. 5e). In contrast, CD69^+^CD103^+^ T cells from untreated and anti-PD-1-treated B16F10E and B16F10E-KO tumours displayed similar percentages of Ki-67^+^ lymphocytes. More importantly, CD8^+^ TIL from B16F10E-KO was much more strongly enriched with granzyme B^+^ T cells than B16F10E untreated or treated with anti-PD-1, with no further enrichment after PD-1 blockade in the former tumours (Fig. 5f). Anti-PD-1-treated-B16F10E-KO-derived CD8^+^ T cells were also more enriched with CD69^+^CD103^+^ TIL than CD8^+^ T cells from isotype-treated B16F10E-KO and B16F10E treated with anti-PD-1. Moreover, CD69^+^CD103^+^CD8^+^ T cells from anti-PD-1-treated B16F10E-KO tumours expressed more frequently granzyme B than isotype-treated B16F10E-KO (Fig. 5f). Accordingly, CD8^+^ TIL from anti-PD-1-treated B16F10E-KO was able to kill the cognate tumour target slightly more than CD8^+^ TIL from isotype-treated B16F10E-KO (Fig. 5g). Cytotoxicity correlated with upregulation of MHC-I and PD-L1 molecules on anti-PD-1-treated B16F10E-KO tumour cells as compared to isotype-treated B16F10E-KO (Supplementary Fig. 7c), which correlated with high production of IFNγ by CD8^+^ TIL (Supplementary Fig. 7d). However, a comparison between anti-PD-1-treated B16F10E and anti-PD-1-treated B16F10E-KO for the expression of MHC-I and PD-L1 did not reveal significant differences suggesting that the observed CTL-mediated activities in α_V_-KO tumours were not associated with optimized antigen presentation by target cells nor with decreased PD-L1 expression. These results suggest that decreased tumour α_V_ expression and concomitant inhibition of autocrine TGF-β maturation result in recruitment and activation of tumour-reactive CD8^+^ T cells.

### CD8^+^ T_RM_ cells are involved in anti-PD-1 response of α_V_-KO tumours

**Fig. 6:**
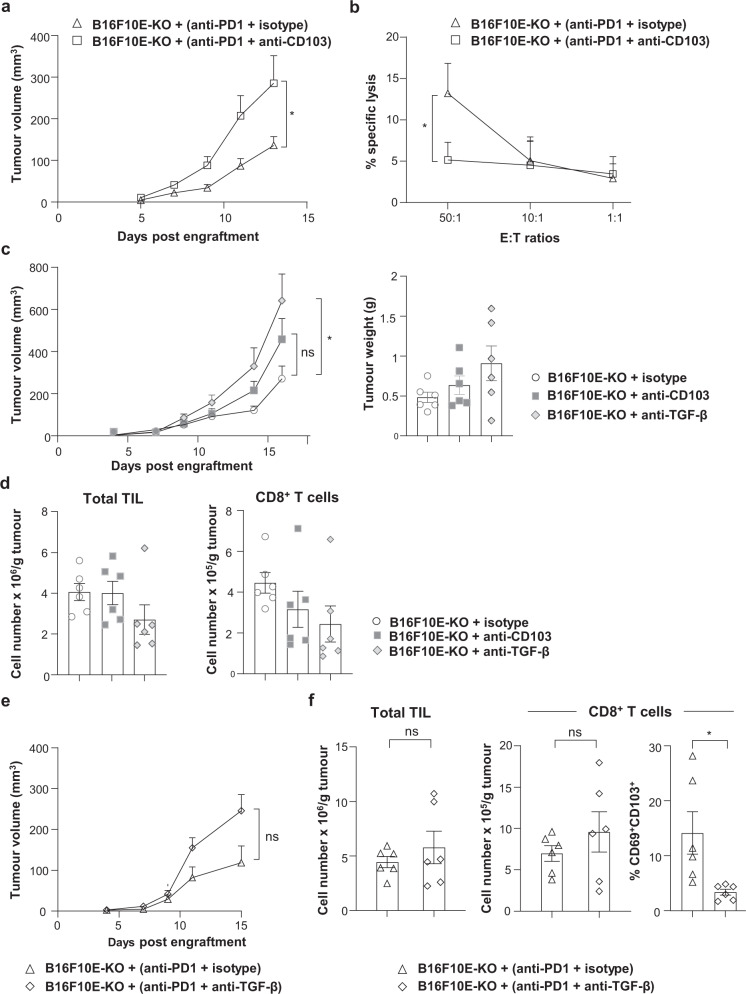
Effect of anti-CD103 and TGF-β neutralizing mAb on tumour growth and PD-1 blockade therapy in B16F10E-KO tumours. **a** Neutralizing anti-CD103 mAb inhibits the effect of anti-PD-1. Growth of B16F10E-KO tumours, treated with anti-PD-1 and then receiving anti-CD103 blocking mAb (i.t.) or isotype control. Tumour volumes are given as means ± SEM of six mice/group (*p = 0.043). Data are from two independent experiments out of two. **b** Cytotoxic activity of CD8^+^ TIL isolated from B16F10E-KO tumours (*n* = 3) treated with anti-PD-1 (i.p.) plus isotype control (i.t.) or anti-PD-1 plus anti-CD103 (i.t.). Cytotoxicity against B16F10E-KO tumour cells was determined by a conventional ^51^Cr release assay at indicated E:T ratios. Data are from one independent experiment out of two (*p = 0.030). **c** Effects of anti-TGF-β and anti-CD103 neutralizing mAb on tumour growth. Mice were engrafted with B16F10E-KO and then treated with anti-TGF-β, anti-CD103, or isotype control (i.t.). Tumour volumes are given as means ± SEM of six mice/group. Right, tumour weights of B16F10E-KO (*n* = 6) recovered at day 17 (*p = 0.024). **d** Left, absolute cell counts of total TIL and right, CD8^+^ T cells infiltrating B16F10E-KO tumours treated with anti-TGF-β, anti-CD103, or isotype control (*n* = 6). **e** Effects of anti-TGF-β neutralizing mAb on PD-1 blockade. Growth of B16F10E-KO tumours, treated with anti-PD-1 and then received anti-TGF-β blocking mAb (i.t.) or isotype control. Tumour volumes are given as means ± SEM of six mice/group (*p* = 0.196). **f** Left, absolute counts of total TIL in B16F10E-KO tumours treated with anti-PD-1 plus anti-TGF-β or anti-PD-1 plus isotype control (*p* = 0.418). Middle panel, absolute counts of CD8^+^ T cells (*p* = 0.343) and right, percentages of CD69^+^CD103^+^ (*p = 0.020) among CD8^+^ T cells from B16F10E-KO tumours treated with anti-PD-1 mAb (i.p.) plus anti-TGF-β (i.t.) or anti-PD-1 plus isotype control (*n* = 6). Each symbol represents an individual tumour; horizontal lines correspond to mean ± SEM (**c** right, **d**, **f**). Data were calculated with unpaired *t*-test (**a**−**c**, **e**, **f**) and one-way ANOVA with Tukey’s correction (**c** right, **d**.). Source data are provided as a Source Data file.

The above studies suggest that CD103^+^CD8^+^ T cells are implicated in response to anti-PD-1 and that tumour α_V_-knockout does not eradicate their formation at the tumour site. To test this hypothesis, mice were engrafted with B16F10E-KO, treated with anti-PD-1, then received anti-CD103 blocking mAb or isotype control, and followed for tumour development. Results indicated that the beneficial effect of PD-1 blockade was inhibited when mice received intra-tumoural injection of anti-CD103 (Fig. 6a). This inhibition was associated with a decrease in target cell killing by CD8^+^ TIL from anti-PD-1 plus anti-CD103-treated B16F10E-KO as compared to CD8^+^ TIL from anti-PD-1 plus isotype-treated tumours (Fig. 6b). Notably, anti-CD103 alone had no effect on tumour growth and weight of B16F10E-KO (Fig. 6c) and B16F10E (Supplementary Fig. 7e). Anti-CD103 alone had also no effect on the number of TIL and CD8^+^ T cells in B16F10E-KO (Fig. 6d) and B16F10E (Supplementary Fig. 7f) and on the percentage of conventional (c)DC and CD103^+^ cDC in both tumours (Supplementary Fig. 7g, h). These data suggest that a CD103^+^CD8^+^ TIL subset is involved in tumour growth control and that this subset is differentiated in the TME independently of tumour α_V_-mediated TGF-β activation.

To test whether TGF-β is mandatory for the differentiation of CD103^+^CD8^+^ T cells in the tumour and response to PD-1 blockade, B16F10E-KO-engrafted mice were treated with anti-PD-1, received intra-tumoural injection of neutralizing anti-TGF-β mAb or isotype control, and monitored for tumour progression. Results showed that a combination of anti-PD-1 plus anti-TGF-β had only a marginal effect on tumour growth (Fig. 6e) and infiltration by TIL and CD8^+^ T cells, even though a decrease in the percentages of CD103^+^CD69^+^CD8^+^ T cells was observed (Fig. 6f). Notably, anti-TGF-β alone had also no effect on B16F10E-KO tumour weight as compared to isotype control, and an increase in tumour growth was even observed (Fig. 6c). No effect was also observed on B16F10E tumour growth and weight (Supplementary Fig. 7e), and on the number of TIL and CD8^+^ T cells in B16F10E-KO (Fig. 6d), the number of TIL in B16F10E (Supplementary Fig. 7f), and on the percentage of cDC and CD103^+^ cDC infiltrating both tumours (Supplementary Fig. 7g, h). These data support the observation that CD8^+^ T_RM_ cells play an important role in response to anti-PD-1 and that TGF-β is involved in their formation in a cancer-cell-α_V_-integrin-independent manner.

## Discussion

In this report, we show that α_V_ integrin is frequently expressed by NSCLC tumours. This integrin is produced by multiple cell types, ranging from immune cells to cancer cells, and it is known to be a key activator of LAP-TGF-β^32^. A critical role for α_V_ integrin family members, including α_V_β_6_ and α_V_β_8_, has been highlighted in regulating tissue and immune homeostasis by activating TGF-β. In this respect, it has been reported that fibroblast α_V_ regulates inflammation and fibrosis in the lung by a mechanism involving TGF-β activation^33^. Integrin α_V_-mediated activation of TGF-β also promotes tumour progression through multiple pathways including epithelial-to-mesenchymal transition, an increase of angiogenesis and invasion, formation of cancer-associated fibroblasts, and suppression of T cell-mediated immune surveillance^34,35^. Consistently, we show here that tumour α_V_ interacts and activates autocrine TGF-β and that its knockout inhibits activation of the cytokine and reshapes the phenotypic and functional proprieties of CD8^+^ TIL. This inhibition is incomplete emphasizing that additional mechanisms, such as MMP14 on cancer cells and α_V_ integrin on infiltrating immune cells, including CD8^+^ and CD4^+^ TIL, and DC^28,36^, can activate latent TGF-β within the TME.

Targeting tumour α_V_ has been proposed as a strategy for cancer treatment. In this regard, blocking α_V_β_8_ and subsequent TGF-β activation has been shown to inhibit tumour growth by a mechanism independent of PD-1/PD-L1^27^. Therefore, multiple potential therapeutic agents, such as α_V_-RGD inhibitors and tumour-homing peptide (iRGD), have been developed for treating neoplasms, including lung cancer^32,37,38^. Targeting the *ITGAV* gene has also been used as a therapeutic approach for metastatic cancer^39^. Notably, antagonists of α_V_ observed promising preclinical results with anti-angiogenic and anti-metastatic effects^40^. However, most of the clinical trials with inhibitors targeting α_V_ integrin failed to demonstrate therapeutic benefits^32^. Along the same lines, our data indicate that decreased expression of α_V_ in lung tumours had no effect on survival in treatment-naïve NSCLC patients. In addition, although tumour α_V_-knockout greatly increased tumour infiltration by CD8 T lymphocytes in our mouse model, it failed to delay tumour progression. Indeed, our in vivo studies showed similar growth kinetics of α_V_-proficient and -deficient tumours even though the latter were much more strongly infiltrated by proliferative PD-1^+^CD8^+^ effector T cells. Thus, it is clear that targeting tumour α_V_ as single therapeutic agent is not sufficient in the clinical setting to reliably eradicate cancer. TGF-β inhibitors as single therapeutic agents also observed limited clinical success, likely due to restricted targeting of TGF-β-responsive cells and inadequate effects on cancer cell proliferation^41^. Consistently, targeting TGF-β with neutralizing mAb had no effect on B16F10E tumour growth. Moreover, the development of cancer therapies targeting TGF-β signalling has been hindered by dose-limiting toxicities^42^. Thus, targeting tumour α_V_ in combination with ICB appears a promising strategy to selectively target non-immune cells and ensure a superior safety profile compared to TGF-β inhibitors.

Tumour α_V_ integrin is considered as a serious cause of resistance to ICB by activating TGF-β and thereby excluding CD8^+^ T cells from the TME. Therefore, manipulating α_V_ seems a promising approach to improve the clinical success of ICB. In this context, we show that decreased expression of tumour α_V_ correlates with improved outcome in NSCLC patients treated with anti-PD-(L)1. Our data indicate that α_V_^low^ tumour-bearing patients displayed improved PFS compared to α_V_^high^ tumour-bearing patients associated with an increase in tumour infiltration by CD103^+^CD8^+^ lymphocytes. Moreover, our in vivo mouse model revealed that tumour α_V_ knockout combined with anti-PD-1 resulted in delayed tumour progression likely associated with decreased TGF-β activation and concomitant T cell recruitment. Consistently, blockade of α_V_β_8_ and PD-1 synergistically promoted better anti-tumour response and more beneficial effects^27^. The present study further shows that beneficial effects are likely associated with improved CD8 T cell immunity and increased tumour infiltration by tumour-specific CD103^+^CD69^+^ T_RM_ cells. Indeed, tumour growth control was dependent on anti-tumour CTL response and correlated with an increase in tumour infiltration by proliferative tumour-reactive CD8^+^ T lymphocytes producing granzyme B. CD8^+^ T cell-mediated cytotoxicity against the cognate target correlated with upregulation of MHC-I and PD-L1 on B16F10E-KO cells, excluding an association of the beneficial effect with a reduced PD-L1 expression on α_V_-knockout tumours as previously reported^43^. Of note, α_V_-deficient cancer cells displayed dramatic morphological modifications probably due to alteration in cell-adhesive capacities. However, these changes did not affect tumour cell susceptibility to CTL-mediated killing in vitro, suggesting that in vivo α_V_ modulation would not have an impact on target cell sensitivity to CD8^+^ T cells. Preclinical mouse models revealed that TGF-β blockade also unleashed an anti-tumour CTL response, rendering tumours more susceptible to anti-PD-(L)1^22^. Thus, co-administration of blocking anti-TGF-β and anti-PD-L1 antibodies facilitated T cell recruitment and promoted anti-tumour immunity leading to tumour regression^44^. Selective targeting of LAP-TGF-β overcomes primary resistance to ICB by increasing intra-tumoural CD8^+^ T cells and decreasing immunosuppressive myeloid cells^42^. Moreover, simultaneous targeting of TGF-β and PD-L1 has been described to enhance anti-tumour activity by increasing the influx and functionality of CD8^+^ T lymphocytes in the TME^45,46^. However, targeting TGF-β and PD-1 with specific blocking antibodies had no effect in our in vivo mouse model, suggesting that a minimal level of the cytokine is needed for T_RM_ cell formation and control of tumour progression.

TGF-β is an important regulator of local immune responses via its participation in the differentiation of CD103^+^CD8^+^ T_RM_ cells in peripheral tissues and solid tumours, at least in part by triggering CD103 expression. Along the same lines, we show that by activating TGF-β, tumour α_V_ promotes CD103 expression on activated CD8^+^ T lymphocytes in vitro, and that integrin knockout inhibits this process at least in part, by compromising activation of Smad2/3, critical regulators of the *ITGAE* gene, which encodes CD103^47^. Although α_V_ knockout induced a slight decrease in the frequency of CD103^+^CD8^+^ TIL in anti-PD-1-treated mice, an increase in the percentage of CD103^+^CD69^+^CD8^+^ T_RM_ cells was observed. These data suggest that by inhibiting TGF-β activation, α_V_ knockout in anti-PD-1-treated tumours results in recruitment and expansion of CD8^+^ T cells, and subsequent induction of CD103 on TCR-engaged CTL as we previously reported^48^. These CD103^+^CD69^+^CD8^+^ T_RM_ cells may correspond to truly tumour-reactive T cells the differentiation of which is associated with TGF-β that might be activated in the TME by a mechanism dependent of MMP on tumour cells or α_V_ integrins on immune cells. Although tumour α_V_-dependent active TGF-β may participate in CD103^+^CD8^+^ T_RM_ differentiation, higher levels of TGF-β may compromise T cell recruitment in tumours and their expansion at the memory phase. We consistently observed in anti-PD-1-treated NSCLC patients that the density of CD8^+^CD103^+^ T_RM_ cells in tumours with α_V_^low^ profile was higher than in tumours with α_V_^high^.

As opposed to CD103^+^CD8^+^ T cells, our in vivo studies showed a strong decrease in the frequency of CD103^+^FoxP3^+^CD4^+^ Treg in α_V_-knockout tumours, suggesting that CD103 expression in FoxP3^+^CD4^+^ and CD8^+^ T cells is regulated by distinct mechanisms. CD103^+^CD4^+^ Treg were described as suppressing T-effector cell activation more strongly than their CD103^neg^ counterparts, and that silencing TGF-β reduced their frequency and suppressive activity^49^. Targeting LAP on Treg cells also promoted anti-tumour immunity by decreasing TGF-β production and increasing tumour infiltration with activated CTL^50^. Consistent with this, we observed that an optimized local anti-tumour CD8 T cell immunity correlates with a decreased frequency of CD103^+^CD4^+^ Treg cells in anti-PD-1-treated-α_V_-knockout tumours. Blockade of α_V_ on human Treg cells has also been shown to inhibit Treg-mediated immune suppression by inhibiting TGF-β maturation^51,52^. This integrin is also expressed by CD103^+^ DC and confers the capacity to generate Treg by activating TGF-β^53^. Thus, targeting α_V_ integrin in tumour and immune cells would permit inhibition of Treg generation and promote CD8^+^ T cell recruitment and effector functions within the TME.

We previously demonstrated that CD103 is directly involved in T_RM_ recruitment within epithelial tumour regions, and that TGF-β enhanced CD103-dependent T cell adhesion and migration and promoted anti-tumour CTL functions^54^. CD103 also participates in T_RM_ residency in tumours, and targeting this integrin or TGF-β has been shown to decrease the number of intra-tumoural T_RM_ cells^17,55^. CD103 favours T cell retention in epithelial tumour islets through binding to its ligand, the epithelial cell marker E-cadherin^54,56^. Thus, TGF-β is involved in the retention of T_RM_ at least in part through the induction of CD103 and CD69^57^. Our in vivo studies showed that α_V_ knockout combined with PD-1 blockade induced an increase in intra-tumoural granzyme-B-producing CD103^+^CD69^+^CD8^+^ T cells with a parallel optimization of CTL-mediated target cell killing. Moreover, blockade of CD103 inhibited growth control of anti-PD-1-treated-α_V_-knockout tumours associated with a decrease in T cell-mediated cytotoxicity toward target cells. Thus, by activating TGF-β, α_V_ integrin plays a dual role in regulating anti-tumour T cell response; on the one hand by controlling CD8^+^ T_RM_ differentiation and retention in epithelial tumour regions, and on the other, by participating in T cell exclusion and dysfunction. The capacity of α_V_ on stromal cells in controlling the formation and epithelial residence of CD8^+^ T_RM_ cells by activating TGF-β has been demonstrated in healthy tissues^58^. Moreover, the expression of α_V_ by DC has been reported to prepare naïve CD8^+^ T cells for the formation of epithelial T_RM_ cells^36^. These findings support the hypothesis that α_V_ on tumour-infiltrating immune cells participate in the formation of CD103^+^CD8^+^ T_RM_ in the TME and in regulating T cell recruitment and functions.

Overall, we conclude that α_V_ on tumour cells shapes the tumour immune infiltrate and dictates the cellular and cytokine components of the TME by activating TGF-β and thereby by regulating CD8 T cell immunity and response to anti-PD-1. This integrin could be considered as a potential biomarker of response to T cell-based cancer immunotherapies and targeting it may improve the clinical benefit from ICB.

## Methods

### Cohorts of treatment-naïve and anti-PD-(L)1-treated NSCLC patients

The treatment-naïve cohort of early-stage NSCLC patients includes a total of 113 tumour samples^15^. The monocentric retrospective study at Gustave Roussy describes a first cohort (cohort 1) of 106 and a second cohort (cohort 2) of 51 advanced NSCLC patients treated between 2012 and 2020, with anti-PD-(L)1 administered in a variety of settings. Demographic, clinical, pathological, and molecular data were collected (Supplementary Tables 1 and 2). Radiological assessments were performed every 8 weeks per RECIST v1.1 and per the investigator’s discretion. This study was approved by the Institutional Review Board of Gustave Roussy (Commission Scientifique des Essais Thérapeutiques [CSET]) and informed consent from patients was obtained.

### Immunohistochemistry staining

IHC was performed on archived FFPE tumour tissue specimens using Ventana Benchmark and Discovery automated platforms. After deparaffinization and epitope retrieval in CC1 buffer (pH = 8, 36 min at 95 °C), tissue sections were incubated with primary mAb for α_V_ (clone EPR16800, Abcam ab179475, 1/1000) during 1 h at room temperature. Amplification and detection were performed with an Ultraview kit using amplification and 3,3′-diaminobenzidine as a chromogen. Integrin α_V_ staining was evaluated by a pathologist as the prominent intensity for each tumour. Tumours were considered α_V_^low^ when cancer cells were negative for α_V_ expression (α_V_^neg^) or when their α_V_ expression level was weaker than on stromal cells and infiltrating immune cells. They were considered α_V_^high^, when α_V_ expression on more than 75% of cancer cells was stronger than on stromal and immune cells. Multiplexed fluorescent IHC for CD8^+^CD103^+^ and CD8^+^CD103^neg^ lymphocytes was performed by sequential staining of a single tissue section with anti-CD8 (clone SP16, Spring Bioscience M3160, 1/200), anti-CD103 (clone EPR4166-2, Abcam ab129202, 1/200), and anti-cytokeratin (clones AE1/AE3, Dako GA05361-2, 1/100)^14^. For each staining, the HRP-conjugated amplification system was associated with a tyramide-coupled fluorophore: Opal 690, Opal 250, and Opal 570, respectively. Multispectral fluorescent images were captured using the Vectra 3 microscope (PerkinElmer) and regions of interest were selected. Image analysis using InForm software (PerkinElmer) included spectral unmixing, nuclei detection based on DAPI staining, and cell segmentation followed by cell phenotyping for identification of cell populations defined by the combination of individual markers. The density (number of cells per square mm) of CD8^+^, CD8^+^CD103^+^, and CD8^+^CD103^neg^ was determined for each tumour sample in the total tumour area based on tissue segmentation. Results from image analysis were validated for all cases.

### Patient outcome and IHC data analyses

OS for treatment-naïve patients was calculated from the date of primary tumour resection until death due to any cause. PFS for anti-PD-(L)1-treated patients was calculated from the date of the first immunotherapy administration until disease progression or death due to any cause, whichever occurred first. The best cut-point for total CD8^+^, CD8^+^CD103^+^, and CD8^+^CD103^neg^ TIL was assessed using the log-rank maximization method^59^. Survival analyses were performed using the Kaplan−Meier method and the log-rank test. All *p*-values inferior to 0.05 were considered statistically significant.

A Cox proportional hazards regression model was used to evaluate independent prognostic factors for OS and PFS. Variables included in the final multivariate model were selected according to their clinical relevance and statistical significance in univariate analysis (*p*-value cut-off = 0.10). The proportional hazard hypothesis was verified with the Schoenfeld residual method. Predictive factors of disease control were tested with logistic regression in univariate and multivariate analyses. The alpha level was 5%. Statistical analyses were performed with RStudio v1.1.463 (free software environment for statistical computing and graphics).

### TCGA data analysis

Using http://kmplot.com, analysis of gene-expression datasets derived from NSCLC patients was performed on a group of stage I surgically managed patients who did not receive radiotherapy or chemotherapy (*n* = 70). Gene-expression data were automatically computed to generate Kaplan–Meier plots (doi: 10.1371/journal.pone.0082241)^60^.

### Quantitative RT-PCR

Total RNA was immediately extracted from human and murine samples using TRIzol reagent (Invitrogen). cDNA was synthesized with the Maxima First Strand cDNA Synthesis Kit (ThermoFischer Scientific). qRT-PCR was performed on a Step-One Plus (Applied Biosystems) using Maxima SYBR Green Master Mix (ThermoFischer Scientific). Expression levels of transcripts were normalized to *18S* expression. PCR primers and probes for human (*ITGAV*, *ITGB1, ITGB3, ITGB5, ITGB6*, *ITGB8*, *TGFB1*, and *18S*) and mouse (*itgb1, itgb3, itgb5, itgb6*, *itgb8,* and *18S*) genes (Supplementary Table 6) were provided by Sigma-Aldrich and used according to the manufacturer’s recommendations.

### Antibodies and flow cytometry

For human tumour cell surface and intracellular staining, anti-α_V_ (clone NKI-M9, BioLegend 327908, 1/100), -β_6_ (clone 437211, R&D Systems MAB4155, 1/100), -β_8_ (clone 416922, R&D Systems MAB4775, 1/100), anti-E-cadherin (clone 67A4, BioLegend 324106, 1/200) and -EpCAM (clone 9C4, BioLegend 324207, 1/200) mAb were used. Cell surface and intracellular staining of mouse cells was performed on single-cell suspensions using antibodies specific to the following molecules: CD51 (integrin α_V_, clone RMV-7, BioLegend 104106, 1/200), E-cadherin (clone DECMA-1, BioLegend 147307, 1/200), H2-K^b^/H2-D^b^ (clone 28-8-6, BioLegend 114605, 1/200), PD-L1 (clone 10F.9G2, BioLegend 124313, 1/100), integrin β5 (clone KN52, ThermoFischer Scientific 11-0497-41, 1/50), integrin β3 (clone 909114, R&D systems FAB8557A, 1/100), CD3 (clone 17A2, BioLegend 100241, 1/200), CD4 (clone RM4-5, BioLegend 100536, 1/200), FoxP3 (clone FJK-16S, Thermo Ficher 35-5773-80, 1/100), CD8α (clone REA601, Miltenyi 130-109-252, 1/20), CD62L (clone MEL-14, BioLegend 104405, 1/200), CD44 (clone IM7, BioLegend 103030, 1/100), PD-1 (clone 29F.1A12, BioLegend 135223, 1/100), CD69 (clone H1.2F3, BioLegend 104512, 1/100), CD103 (clone 2E7, BioLegend 121418, 1/200), KLRG1 (clone 2F1, BioLegend 138407, 1/100), Ki-67 (clone REA183, Miltenyi 130-120-556, 1/11), IFN-γ (clone XMG1.2 BioLegend 505825, 1/50), granzyme B (clone GB11, BioLegend 515403, 1/50), pSmad2/3 (clone 072-670, BD 562586, 1/20), MHC-II (clone REA528, Miltenyi 130-108-004, 1/20) and CD11c (clone N418, BioLegend 117321, 1/200). Dead cells were excluded using the Live/Dead Fixable Blue Dead Cell Stain Kit (Invitrogen 186684). Staining of MMP14 was performed with specific mAb (clone EP1264Y, Abcam ab51074, 1/200) followed by secondary antibody staining (goat anti-rabbit IgG Abcam ab150077, 1/200).

For intracellular staining, cells were fixed, permeabilized with the FoxP3 staining buffer set according to the manufacturer’s instructions (eBioscience 00-5523-00). Flow cytometry was conducted on an LSR Fortessa (BD) and analyzed using FlowJo software V10 (Tree Star).

### Human PBMC and lung TIL

HD blood samples were collected from the French blood bank (Etablissement Français du Sang (EFS); agreement number N°12/EFS/079. PBMC were isolated by a Ficoll-Hypaque gradient.

NSCLC tumours were obtained from the Centre chirurgical Marie Lannelongue. For freshly isolated TIL and cancer cells, human lung tumours were dissociated mechanically and enzymatically using a Tumour Dissociation Kit (Miltenyi, 130-095-929). Mononuclear cells were then isolated by a Ficoll-Hypaque gradient. Tumour cells were isolated by magnetic separation using Tumour Cell Isolation Kit (Miltenyi, 130-108-339). Healthy donors and patients provided their written informed consent prior to inclusion in this study. All human experiments were approved by the Institutional Review Board of Gustave Roussy.

### Human tumour cell lines and CTL clone

The IGR-B2 cell line and the autologous B90 CTL clone were derived from patient Bla large cell carcinoma^30^. The allogeneic NSCLC cell lines IGR-Pub, IGR-Heu, ADC-Coco, ADC-Tor, and ADC-Let were derived from tumour specimens in one of our laboratories. H1355 (adenocarcinoma), H460, and H1155 (large cell carcinoma) were a generous gift from Dr. S. Rogers (Brigham and Women’s Hospital, Boston, MA), and A549 (adenocarcinoma), SK-Mes, Ludlu (squamous cell carcinoma, SCC) and DMS53 (small-cell-lung carcinoma, SCLC) were purchased from the European Collection of Cell Cultures^30^. Lung tumour cells lines were grown in DMEM/F-12 medium (ThermoFischer Scientific) supplemented with 10% fetal calf serum (FCS), 1% Ultroser G (Pall), 1 mM sodium pyruvate, and antibiotics (50 U/ml penicillin and 50 μg/ml streptomycin). All the cell lines are mycoplasma-free and were regularly tested for mycoplasma contamination. We regularly authenticate IGR-B2 cell line by testing recognition by autologous B90 CTL clone, and HLA-A2 expression.

### Transfection of IGR-B2 with TGF-β and knockout of α_V_ subunit

The IGR-B2 NSCLC cell line was stably transfected with pcDNA3.1-hTGFβ-LAP plasmid (from one of our laboratories) using Lipofectamine 2000 (ThermoFischer Scientific) according to the manufacturer’s instructions. The cell line obtained was cloned, and one clone, IGR-B2T, was selected for its capacity to produce TGF-β detected in cell supernatant by ELISA (DuoSet, ThermoFischer Scientific).

Homozygous knockout of α_V_ expression in the IGR-B2T cell clone was performed using CRISPR-Cas9 technology. The cells were double transfected with integrin α_V_ CRISPR-Cas9 KO plasmid (Santa Cruz Biotechnology, sc-400506) and integrin α_V_ HDR plasmid (Santa Cruz Biotechnology, sc-400506-HDR). The transfection efficiency was confirmed by flow cytometry for double GFP and RFP expression. The GFP^+^RFP^+^ cells were cell sorted and treated with puromycin (10 µg/ml, Sigma-Aldrich). The generated cell line was tested for α_V_ knockout by FACS, and then cloned by cell sorting. A clone, thereafter named IGR-B2T-KO, was retained for further studies.

### B16F10 cell line and α_V_ knockout clone

The B16F10 melanoma cell line (H-2^b^) was purchased from the American Type Culture Collection (ATCC CRL-6475) and grown in DMEM-F12 medium supplemented with 10% FCS, 2 mM L-glutamine, 1 mM sodium pyruvate, and antibiotics. It was stably transfected with pcDNA-E-cadherin plasmid (gift from Lionel Larue, Institut Curie, Orsay) and a clone, named B16F10E, was isolated and used in all experiments. Homozygous knockout of α_V_ expression was performed as above using the CRISPR-Cas9 technology (sc-421169 and sc-421169-HDR), and a clone, named B16F10E-KO, was selected for further experiments.

### Quantification of TGF-β in conditioned media and tumours

Human and mouse tumour cells were cultured in complete medium, and for an additional 24 h in serum-free medium. The CM was then harvested and concentrated 75× using Amicon Ultra Centrifugal Filters-10 K (Merck). CM was used to measure the concentration of total TGF-β by ELISA using human and mouse TGF-β DuoSet ELISA kits (DY240 and DY1679, ThermoFischer Scientific). For MMP activity inhibition, tumour cells were cultured in the presence of the MMP inhibitor GM6001 (Millipore, CC1010, 1/1000) or DMSO control, and four days later, CM was harvested.

Activation of LAP-TGF-β by parental and α_V_-knockout tumour cells was evaluated using the thymic mink lung epithelial cells Mu.1LV (gift from Céline Prunier, Centre de Recherche Saint Antoine, Paris), cultured with 24 h CM. First, Mu.1LV cells were resuspended in DMEM/F12 medium supplemented with 10% FCS, plated for 24 h, and then transfected with the pGL3-(CAGA)9-Lux reporter construct, which contains concatemerized CAGA elements that bind Smad3 and Smad4 complexes and are transactivated by both TGF-β and Smad3/4 expression^61^. Twenty-four hours after Mu.1LV transfection, CM was added to serum-free Mu.1LV cells, and cells were lysed and assayed for luciferase activity using Bright Glo Luciferase Assay System (Promega). For quantification of active and total TGF-β in mouse B16F10E and B16F10E-KO, tumours were crushed and rinsed with phosphate-buffered saline (PBS), and the solution was used for active and total TGF-β quantification (DY1679 DuoSet ELISA kit, ThermoFischer Scientific).

### Induction of CD103 on activated human CD8 T cells

Plates were coated with 1 µg/ml of anti-CD3 blocking mAb (clone OKT3, BioLegend 317301, 1/1000) for 24 h at 4 °C. 5 × 10^5^ PBMC/well were added and cultured for 3 days at 37 °C and 5% CO_2_ with CM from B16F10E or B16F10E-KO and IGR-B2T or IGR-B2T-KO cells in the absence or presence of a low (1 ng/ml) or high (5 ng/ml) dose of rTGF-β (240-B-002, R&D Systems) or blocking anti-TGF-β mAb (clone 1D11.16.8, BioXcell BE0057, 10 μg/ml). Expression of CD103 (clone Ber-ACT8, BioLegend 350204, 1/100) and KLRG1 (clone 13F12F2, ThermoFicher 12-9488-42, 1/100) on CD3^+^CD8^+^ cells (CD3: clone UCHT1, BioLegend 300463, 1/200; CD8α: clone RPA-T8, BioLegend 301012, 1/200) was then determined by flow cytometry.

### PLA of α_V_ integrin and LAP-TGF-β interaction

The green PLA signal was observed for α_V_ and LAP-TGF-β interaction. PLA was performed on fixed and permeabilized tumour cells according to the manufacturers’ instructions (DuoLink In Situ Detection Reagents Green, Sigma Aldrich). After blocking, anti-α_V_ (clone EPR16800, Abcam ab179475, 1/100) and anti-LAP-TGF-β (clone TW7-16B4, BioLegend 141402, 1/100) antibodies were used. PLA-minus and PLA-plus probes, containing the secondary antibodies conjugated with oligonucleotides (dilution in Antibody diluent 1/5), were added and incubated for 1 h at 37 °C. After hybridization, oligonucleotide ligation and a rolling circular amplification were performed. Cell membranes were stained with WGA red conjugate (Thermo Fisher Scientific W21405, 1/1000), nuclei with Fluoromount-G mounting media containing DAPI (eBioscience), and then analyzed with a confocal microscope SP8 (Leica). The number of PLA signals, marked as green dots on the cell surface, was counted in 25−30 fields containing 500−600 cells, by image analysis (Icy, http://icy.bioimageanalysis.org/download/, v2).

### In vitro cell proliferation assay

Tumour cells were labelled with 5 µM Carboxyfluorescein Diacetate Succinimidyl Ester (CFSE) dye (Stemcell Technologies) for 5 min at 37 °C. Cells were then washed twice with FCS-free medium and cultured for 6 days. The proliferation index was calculated every other day by flow cytometric analysis using ModFit LT™ programme v5.0.

### In vivo experiments

Animal experiments were performed in accordance with Gustave Roussy’s relevant ethical regulations for animal testing and research. This study received ethical approval from the institutional animal committee (CEEA no. 026: 2018-056-16280) after receiving the legal approval from French Minister of Higher Education, Research and Innovation under the procedure number APAFIS#16281-2018072515064652v2. Female C57BL/6 mice were purchased from Envigo. The female athymic nude Crl:NU(NCr)-Foxn1^nu^ mice (6 weeks old) were inbred in-house. For each experiment, groups of four to eight mice, 7−9 weeks of age received 2 × 10^5^ tumour cells subcutaneously in the right flank. Tumour volume was measured using a calliper every second day and estimated using the following formula: π/6 × length × width × thickness (mm^3^). The mice were sacrificed when the tumour size exceeded the acceptable institutional limit of 2000 mM^3^ by CO_2_ inhalation.

For TIL isolation, tumours were harvested at day 14 and digested for 30 min at 37 °C according to the Tumour Dissociation kit protocol (130-096-730, Miltenyi). Tumours were crushed on 100 μm cell strainers and washed twice with PBS 2% FCS. Single-cell suspensions were enriched for CD45^+^ or CD8^+^ cells using anti-CD45 or anti-CD8 microbeads (130-052-301 or 130-117-044, Miltenyi), and then purified using the POSSEL2 programme on MultiMACS. The positive fraction was recovered for TIL analysis and the negative fraction for tumour cell analysis by flow cytometry and ex vivo cytotoxicity assay.

For in vivo blockade with neutralizing mAb, mice received i.p. 100 μg/mouse of anti-CD8 (Bio-X-Cell BE0061; clone 2.43), intra-tumoural (i.t.) 50 μg/mouse of anti-CD103 (Bio-X-Cell BE0026; clone M290), i.t. 25 μg/mouse of anti-TGF-β (Bio-X-Cell BE0057; clone 1D11.16.8) and/or 200 μg/mouse of anti-PD-1 (Bio-X-Cell BE0146; clone RMP1-14) mAb or isotype control (Bio-X-Cell BE0089: IgG2a, clone 2A3; Bio-X-Cell BE0090: IgG2b, clone LTF-2; Bio-X-Cell BE0083: IgG1, clone MOPC-21). For tumour outgrowth experiments with anti-PD-1, mice were treated on days 4, 7, and 11 after tumour inoculation, whereas with anti-CD8 and/or anti-PD-1, mice were treated on days 4, 7, 10, and 13. For experiments with anti-CD103 and/or anti-PD-1, mice were treated on days 4, 7, 11, and 15, whereas for experiments with anti-TGF-β and/or anti-PD-1, mice were treated on days 4, 8, 11, and 14. TIL were sorted and analyzed on day 14 or 15. For experiments with anti-CD103, anti-TGF-β, and/or anti-PD-1, mice were treated on days 4, 7, 11, and 14. TIL were sorted and analyzed on day 15 or 17.

### In vitro cytotoxicity experiments

The cytotoxic activity of B90 CTL clone against autologous IGR-B2, IGR-B2T, and IGR-B2T-KO tumour cells was measured using a conventional 4-h chromium (^51^Cr) release assay^30^. For cytotoxicity with mouse TIL, CD45^+^ cells were isolated and cultivated overnight in a medium containing 50 U IL-2. CD8^+^ T cells were purified using microbeads and co-cultured for 4 h with syngeneic ^51^Cr-labelled B16F10E or B16F10E-KO tumour cells.

### Statistical analysis

Statistical significance was determined with the unpaired or paired Student *t*-test, Welch’s *t*-test, Mann−Whitney *t*-test, one-way analysis of variance (ANOVA) test with Tukey’s correction, and two-way ANOVA. Statistical analyses were performed with GraphPad Prism software V8 (GraphPad Software, Inc., San Diego, CA, USA).

### Reporting summary

Further information on research design is available in the Nature Research Reporting Summary linked to this article.

## Supplementary information

Supplementary Information

Reporting Summary

## Data Availability

The authors state that all data generated during this study are included in the article and its Supplementary Information file and are available from the corresponding author upon reasonable request. The data included in this manuscript were not deposited on public websites but are accessible in a supplementary file containing flow cytometry and immunohistochemistry raw data. Source data are provided with this paper. A Reporting Summary for this article is available as a Supplementary file. The use of publicly available data from lung cancer cohorts were consulted on the website https://kmplot.com/analysis/index.php?p=service&cancer=lung, under the specific data product name: KM Plotter—Lung Cancer. Source data are provided with this paper.

## Source data

Source Data
