# Supplementary material for: Integrin-αV-mediated activation of TGF-β regulates anti-tumour CD8 T cell immunity and response to PD-1 blockade
Source: Nat Commun. 2021 Sep 1;12:5209. doi: 10.1038/s41467-021-25322-y (PMC8410945; doi:10.1038/s41467-021-25322-y)
Supplement: Supplementary file 1 — Supplementary Information [file 41467_2021_25322_MOESM1_ESM.pdf]

Supplementary information

Integrin-αv-mediated activation of TGF-β regulates anti-tumour CD8 T-cell immunity and response to PD-1 blockade

Ines Malenica[1], Julien Adam[1†], Stéphanie Corgnac[1†], Laura Mezquita[2‡], Edouard Auclin[3], Isabelle Damei[1], Laetitia Grynszpan[1], Gwendoline Gros[1], Vincent de Montpréville[1,4], David Planchard[2], Nathalie Theret[5], Benjamin Besse[2] and Fathia Mami-Chouaib[1*]

[1] INSERM UMR 1186, Integrative Tumour Immunology and Immunotherapy, Gustave Roussy, Fac. de Médecine - Univ. Paris-Sud, Université Paris-Saclay, 94805, Villejuif, France

[2] Department of Cancer Medicine, Gustave Roussy Cancer Campus, Institut d'Oncologie Thoracique, Gustave Roussy, Université Paris-Saclay, Villejuif

[3] Medical and Thoracic Oncology Department, Hôpital Européen Georges Pompidou, AP-HP, Paris, France

[4] Hôpital Marie-Lannelongue, Service d'Anatomie Pathologique, 92350, Le-Plessis-Robinson, France

[5] Univ Rennes, Inserm, EHESP, Irset-UMR-S1085, Rennes, France

[†] These authors contributed equally: Julian Adam, Stéphanie Corgnac

[‡] Current address, Laboratory of Translational Genomics and Targeted Therapeutics in Solid Tumours, August Pi i Sunyer Biomedical Research Institute (IDIBAPS), Barcelona, Spain; Medical Oncology Department, Hospital Clínic, Barcelona, Spain

*** Corresponding author**: Fathia Mami-Chouaib, INSERM UMR 1186, Gustave Roussy. 39, rue Camille Desmoulins, F-94805 Villejuif. Phone: +33 1 42 11 49 65, Fax: +33 1 42 11 52 88, e-mail: fathia.mami-chouaib@gustaveroussy.fr

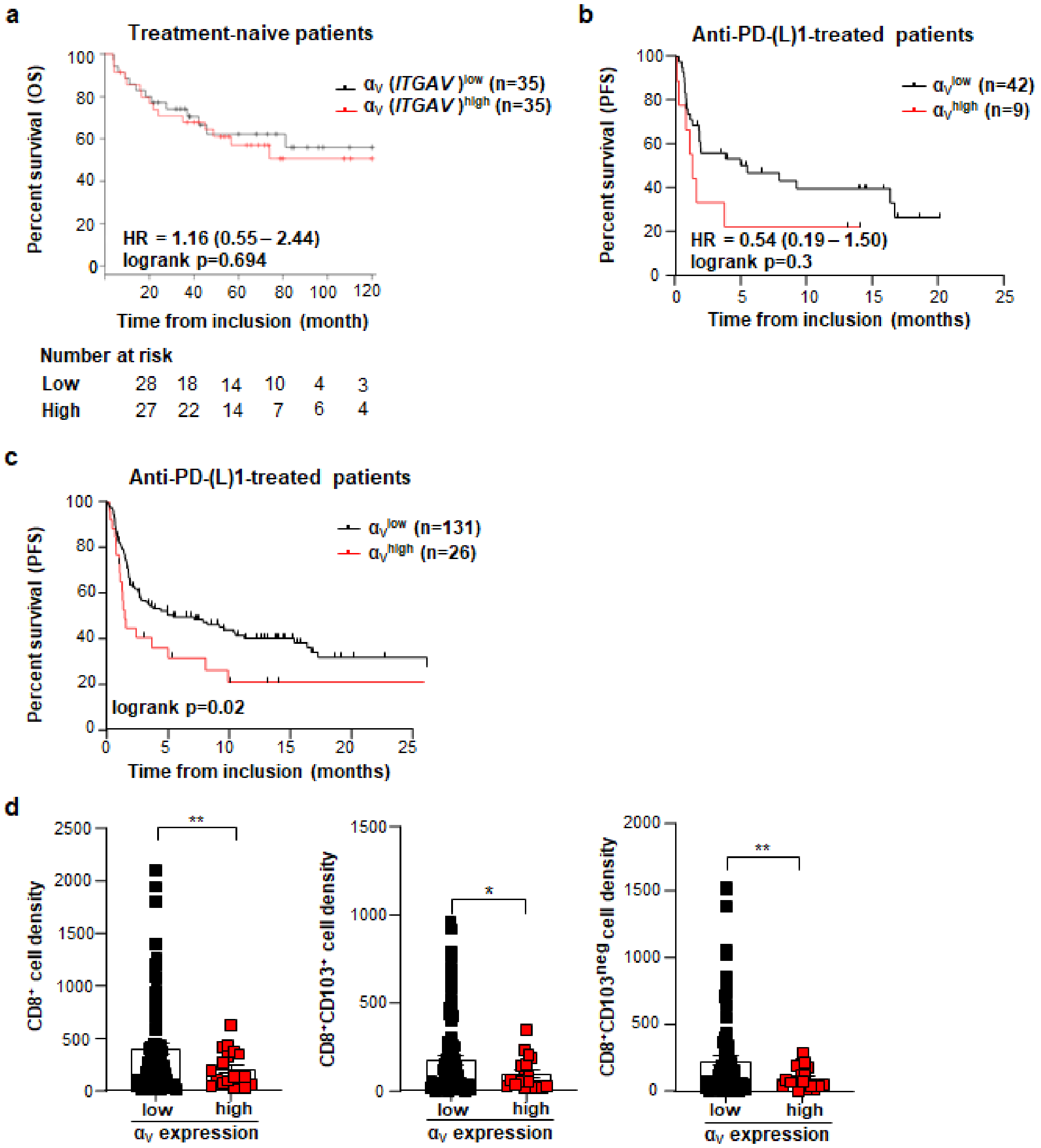

Supplementary Figure 1. Expression of α_V integrin in NSCLC tumours and correlation with patient survival and CD8 T-cell infiltration. **a.** Kaplan-Meier curve shows overall survival (OS) of public TCGA datasets from stage I treatment-naïve lung cancer patients (n=70) according to *ITGAV* mRNA expression. Expression range of the probe 486-8990. Cut-off value=2072. Follow up threshold=120 months. Median survival α_V^{low}= 27.7 months, α_V^{high}= 21.7 months. **b.** Kaplan-Meier curve shows PFS of PD-(L)1 blockade-treated patients (cohort 2) with tumours harbouring low and high expression of α_V integrin; p-value was determined by log-rank test. **c.** Kaplan-Meier curve shows PFS of PD-(L)1 blockade-treated patients (combined cohorts 1 and 2) with tumours harbouring low and high expression for α_V integrin; p-value was determined by log-rank test. **d.** Density of $CD8^+$ (**p=0.004), $CD8^+CD103^+$ (*p=0.024) and $CD8^+CD103^{neg}$ (**p=0.002) cells in α_V^{low} (n=90) and α_V^{high} (n=18) tumours (combined cohorts 1 and 2). Each symbol represents an individual cell type from tumour samples; horizontal lines correspond to mean ± standard error of the mean (SEM) (d.). Data were calculated with log-rank test (a., b. and c.) and Welch's two-sided t test (d.). Footnote, density of $CD8^+$ (p=0.266), $CD8^+CD103^+$ (p=0.542) and $CD8^+CD103^{neg}$ (p=0.352) in α_V^{low} and α_V^{high} tumours from pooled cohorts 1 and 2 using Mann-Whitney t-test. Source data are provided as a Source Data file.

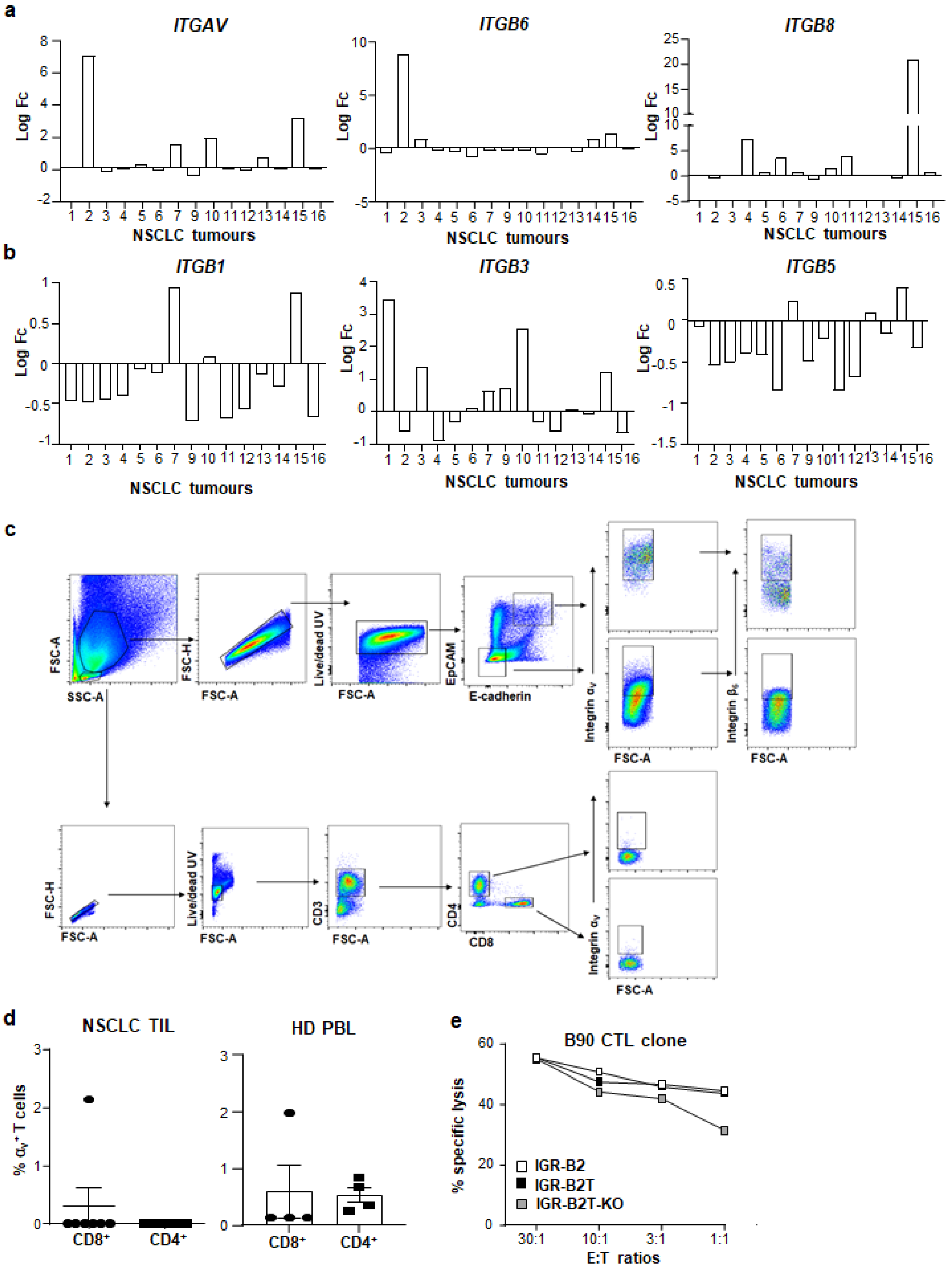

Supplementary Figure 2. Expression of integrin subunits in NSCLC tumours and T cells, and susceptibility of α_V-KO tumour cells to autologous CTL. **a.** Expression of *ITGAV*, *ITGB6* and *ITGB8* transcripts in fresh NSCLC tumours (n=15). **b.** Expression of *ITGB1*, *ITGB3*, *ITGB5* transcripts in human lung tumours. Results were presented on a log scale as a reduction or an increase in gene expression in tumour samples as compared to autologous healthy lung tissues. **c.** Upper panel, gating strategies to determine the percentage of α_V^+ and $\alpha_V^+\beta_6^+$ cells in $EpCAM^+$E-$cadherin^+$ tumour cells and $EpCAM^{neg}$E-$cadherin^{neg}$ non-epithelial cells from freshly resected human NSCLC tumours (used in Fig. 1a and 1b). Lower panel, gating strategies to determine the percentage of α_V^+ cells among $CD4^+$ and $CD8^+$ TIL isolated from fresh NSCLC tumours and human healthy donor (HD) peripheral blood mononuclear cells (PBMC), used in Supplementary Fig. 2d. **d.** Percentages of integrin α_V^+ lymphocytes among $CD8^+$ and $CD4^+$ TIL from human NSCLC (n=7, p=0.377) and HD $CD8^+$ and $CD4^+$ T cells (n=4, p>0.999) isolated from PBMC. Each symbol represents an individual cell type; horizontal line corresponds to mean ± SEM. Data were calculated with unpaired Student t-test. **e.** Cytotoxic activity of the B90 CTL clone toward autologous tumour cells. Cytotoxicity toward IGR-B2, IGR-B2T and IGR-B2T-KO tumour cells was determined by a conventional 4-h ^{51}Cr release assay at indicated E:T ratios. Data shown correspond to one experiment out of two. Source data are provided as a Source Data file.

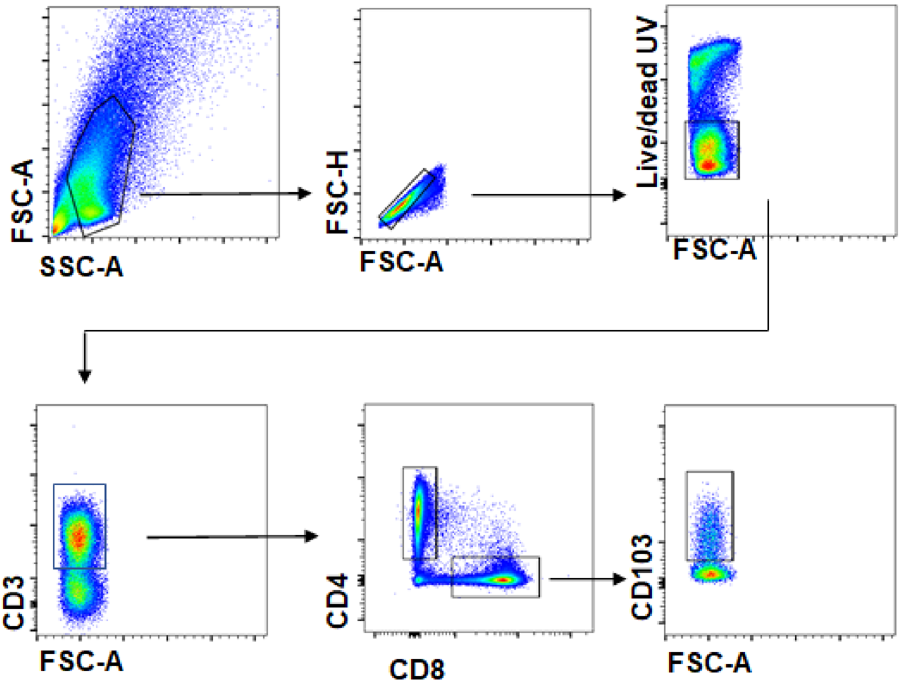

Supplementary Figure 3. Gating strategies for human peripheral blood T cells. Gating strategies to determine the percentage of $CD103^{+}$ cells among $CD8^{+}$ cells from anti-CD3-stimulated healthy donor PBMC. Cells were cultured in the presence of conditioned medium (CM) from IGR-B2T or IGR-B2T-KO cells (used in Fig. 3a-c).

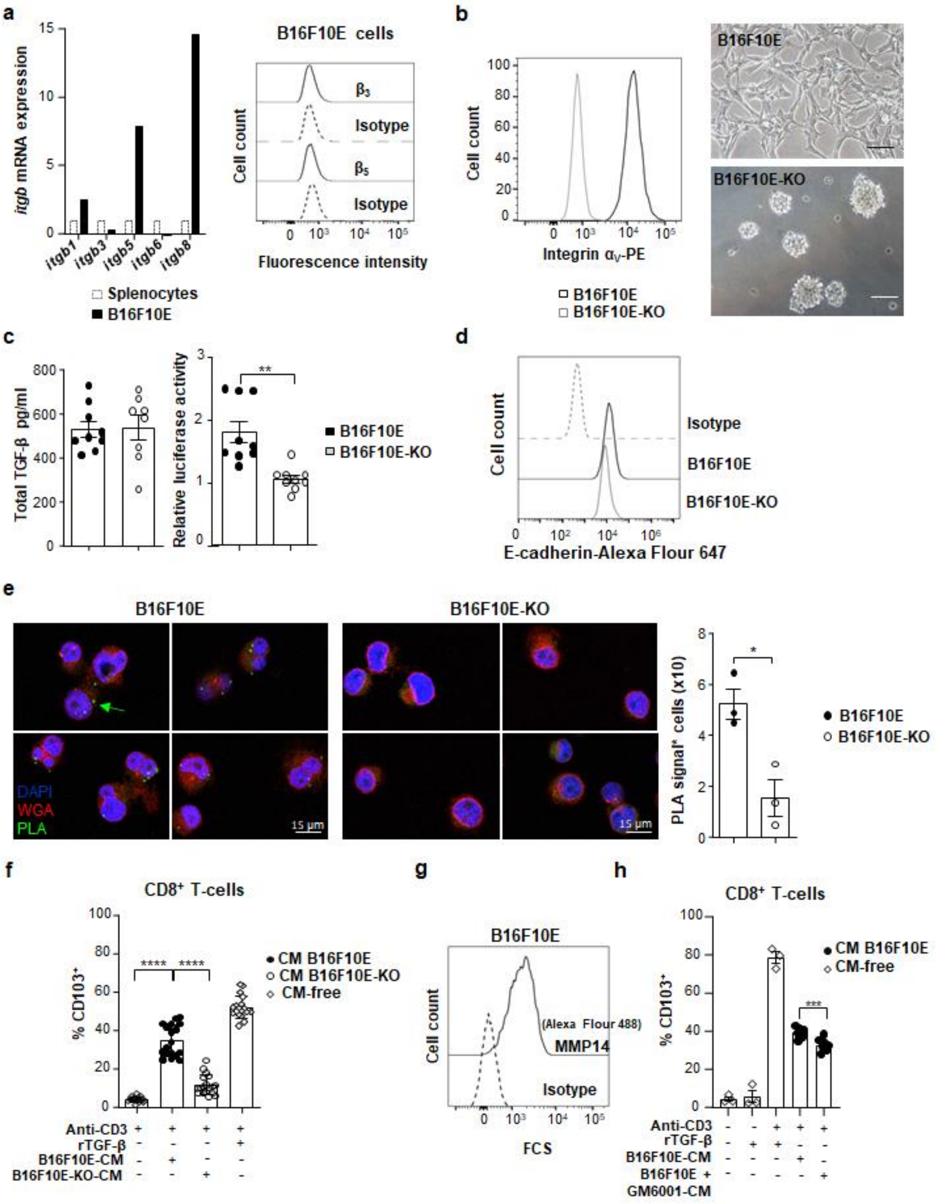

Supplementary Figure 4. Characterisation of murine B16F10E cells and induction of CD103 on CD8$^+$ T cells.
a. Expression of *itgb1, itgb3, itgb5, itgb6,* and *itgb8* transcripts in B16F10E determined by qRT-PCR and normalized to *18S* expression. Control: splenocytes. Right, expression of β_3 and β_5 proteins in B16F10E. **b.** Surface expression of α_V integrin in B16F10E and B16F10E-KO cells. Right, the morphology of B16F10E and B16F10E-KO cells observed by phase-contrast light microscope. Objective: 20x. Scale bar 1 cm. Representative photos are from one experiment out of five. **c.** Concentration of total TGF-β in CM from B16F10E and B16F10E-KO (n=9), measured by ELISA, p=0.917. Right, relative luciferase activity in Mu.1LV reporter cells treated with CM from B16F10E and B16F10E-KO cells (n=9), **p=0.005. Data are normalized to luciferase activity in non-treated cells. Results are represented as mean ± SEM of 9 independent experiments. **d.** Surface expression of E-cadherin on B16F10E and B16F10E-KO (one experiment out of four). **e.** Representative images by confocal microscope of B16F10E and B16F10E-KO. Magnification: 20x. Right, ratio of PLA signal per number of B16F10E and B16F10E-KO cells. Results are means ± SEM of green dots/cell counted in 500-600 cells from 3 independent experiments, *p=0.016.
f. Percentages of CD103$^+$ cells among CD8$^+$ T lymphocytes from PBMC isolated from human HD and stimulated with plastic-coated anti-CD3 mAb alone or in combination with CM from B16F10E or B16F10E-KO (n=18). Positive control: a combination of anti-CD3 plus rTGF-β. Results are represented as mean ± SEM of 18 independent experiments, ****p<0.0001. **g.** Expression of MMP14 on B16F10E cell surface (one experiment out of three).
h. Percentages of CD103$^+$ cells in CD8$^+$ T lymphocytes from HD PBMC (n=8) stimulated with anti-CD3 in combination with CM from B16F10E untreated or treated with the MMP14 inhibitor GM6001, ***p=0.0004. Controls: anti-CD3, rTGF-β and a combination of reagents (n=3). Results are represented as mean ± SEM of 3 independent experiments. Data were calculated with unpaired Student t-test (c., e. and h.) and one-way ANOVA with Tukey's correction (f.). Gating strategy for f. and h. is presented in Supplementary Fig. 3a. Source data are provided as a Source Data file.

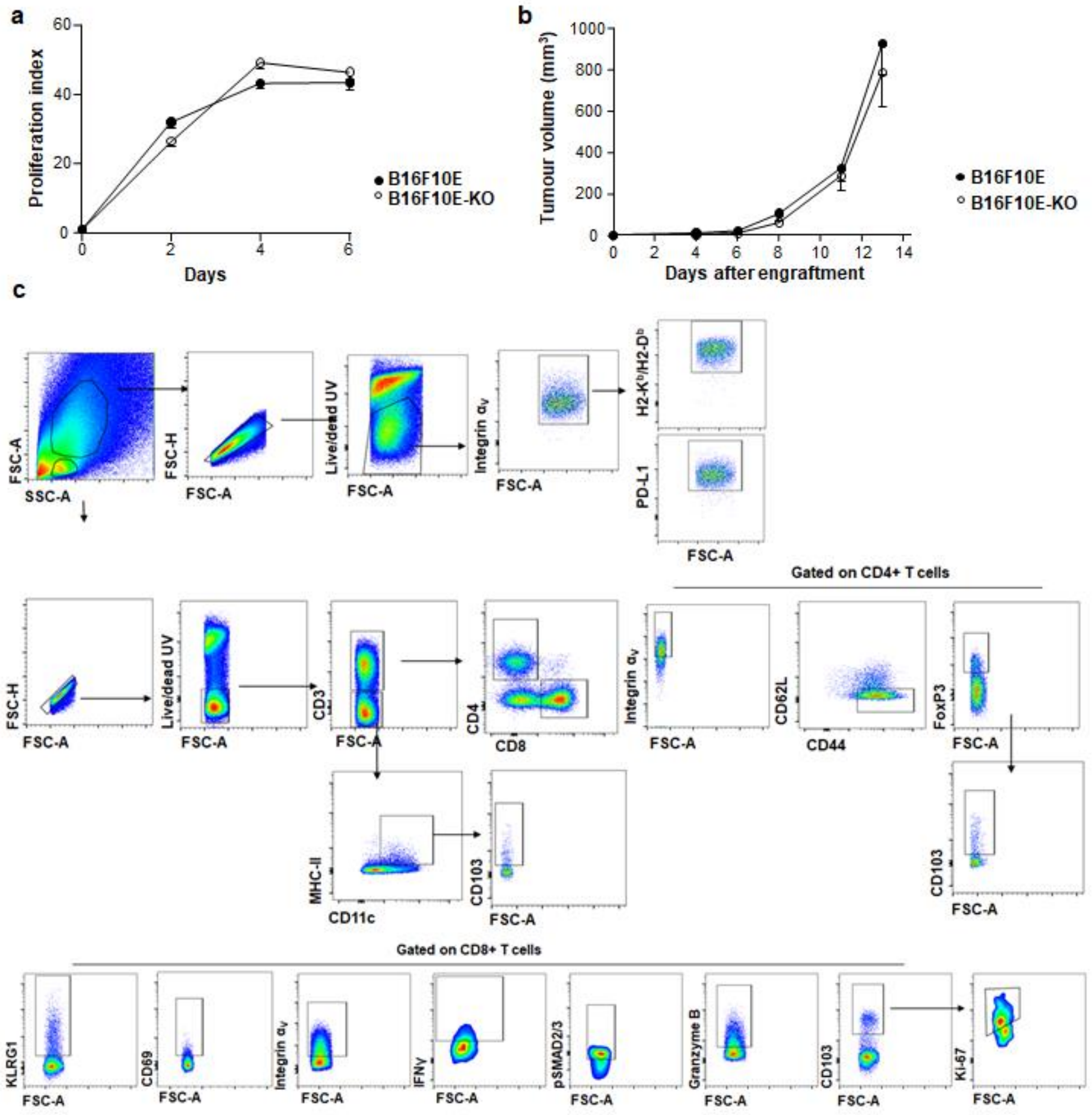

Supplementary Figure 5. B16F10E and B16F10E-KO tumour cell proliferation and growth kinetics, and gating strategies. **a.** Proliferation index of CFSE-labelled B16F10E and B16F10E-KO cells (n=3) analysed in vitro by flow cytometry every two days for six days (p >0,9999). Data are given as means ± SEM of one independent experiment out of three. **b.** In vivo tumour growth. Nude athymic mice were engrafted with B16F10E and B16F10E-KO cells. Tumour volumes are given as means ± SEM of four mice/group (p >0,9999). Mice were sacrificed at day 14, when tumour size exceeded the tolerated institutional limit. **c.** Gating strategy for mouse tumour cells in B16F10E and B16F10E-KO tumours (first upper panel), used in Supplementary Fig.7c. Second till 4th lower row panels, gating strategy for mouse TIL in B16F10E and B16F10E-KO tumours. Representative flow cytometry plots illustrating gating strategy for CD3^{+}CD4^{+} (FoxP3^{neg} and FoxP3^{+}) TIL (2nd row panel), used in Supplementary Fig. 6a and b; and Supplementary 7b. Gating strategy in the 3rd row panel for CD3^{-} TIL, used in Supplementary Fig. 6c and Supplementary Figures 7g-h. For CD3^{+}CD8^{+} TIL, gating strategy is presented in 4th row panel and is used in Fig.4c-g, 5b, d, e and f; 6d and f; Supplementary Figures 6a (right) and d; Supplementary Figures 7a and d. Data were calculated with two-way ANOVA (a. and b.). Source data are provided as a Source Data file.

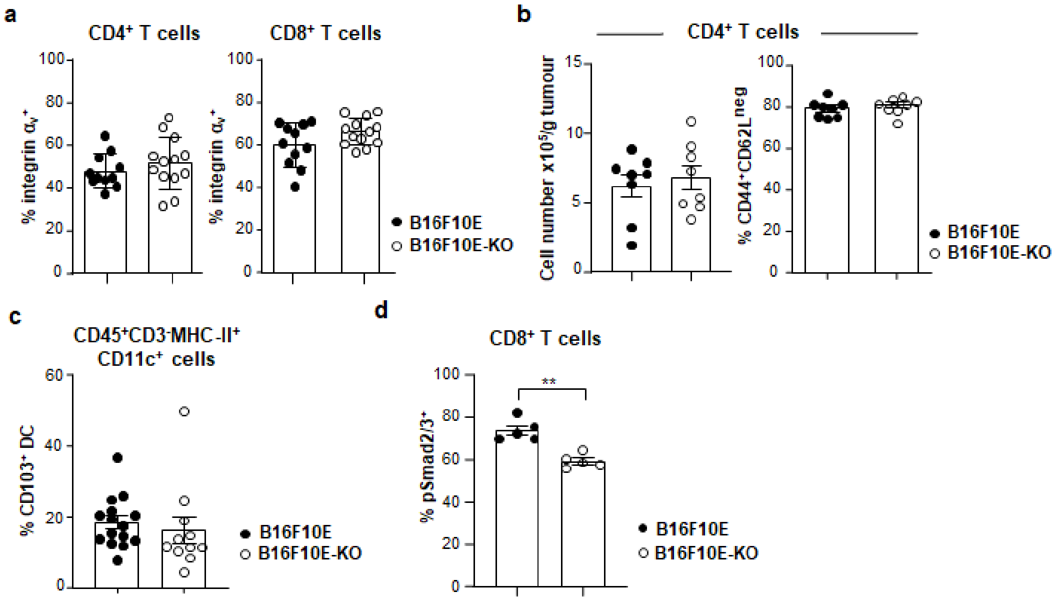

Supplementary Figure 6. B16F10E and B16F10E-KO TIL characterization. **a.** Left, percentages of α_V^+ cells among $CD4^+$ T cells from B16F10E (n=11) and B16F10E-KO tumours (n=13), p=0.384. Right, percentages of α_V^+ cells among $CD8^+$ T cells from the same tumours, p=0.083. Data are from two independent experiments out of three.
b. Left, absolute cell counts of $CD4^+$ T cells from B16F10E and B16F10E-KO tumours (n=8), p=0.641. Right, percentages of $CD44^+CD62L^{neg}$ cells in $CD4^+$ T cells from B16F10E and B16F10E-KO tumours (n=8), p=0.533. Data are from one independent experiment out of three. **c.** Percentages of $CD103^+$ cells among $CD45^+CD3^-MHC\text{-}II^+CD11c^+$ dendritic cells (DC) from B16F10E (n=15) and B16F10E-KO (n=11) tumours, p=0.590. Data are from two independent experiments out of three. **d.** Percentages of pSmad2/3$^+$ cells among $CD8^+$ TIL from B16F10E and B16F10E-KO tumours (n=5), **p=0.004. Data are from one independent experiment out of two. Each symbol represents an individual cell type (a.-d.). Horizontal lines correspond to mean ± SEM (a.-d.). Data were calculated with unpaired Student t-test (a.-d.). Source data are provided as a Source Data file.

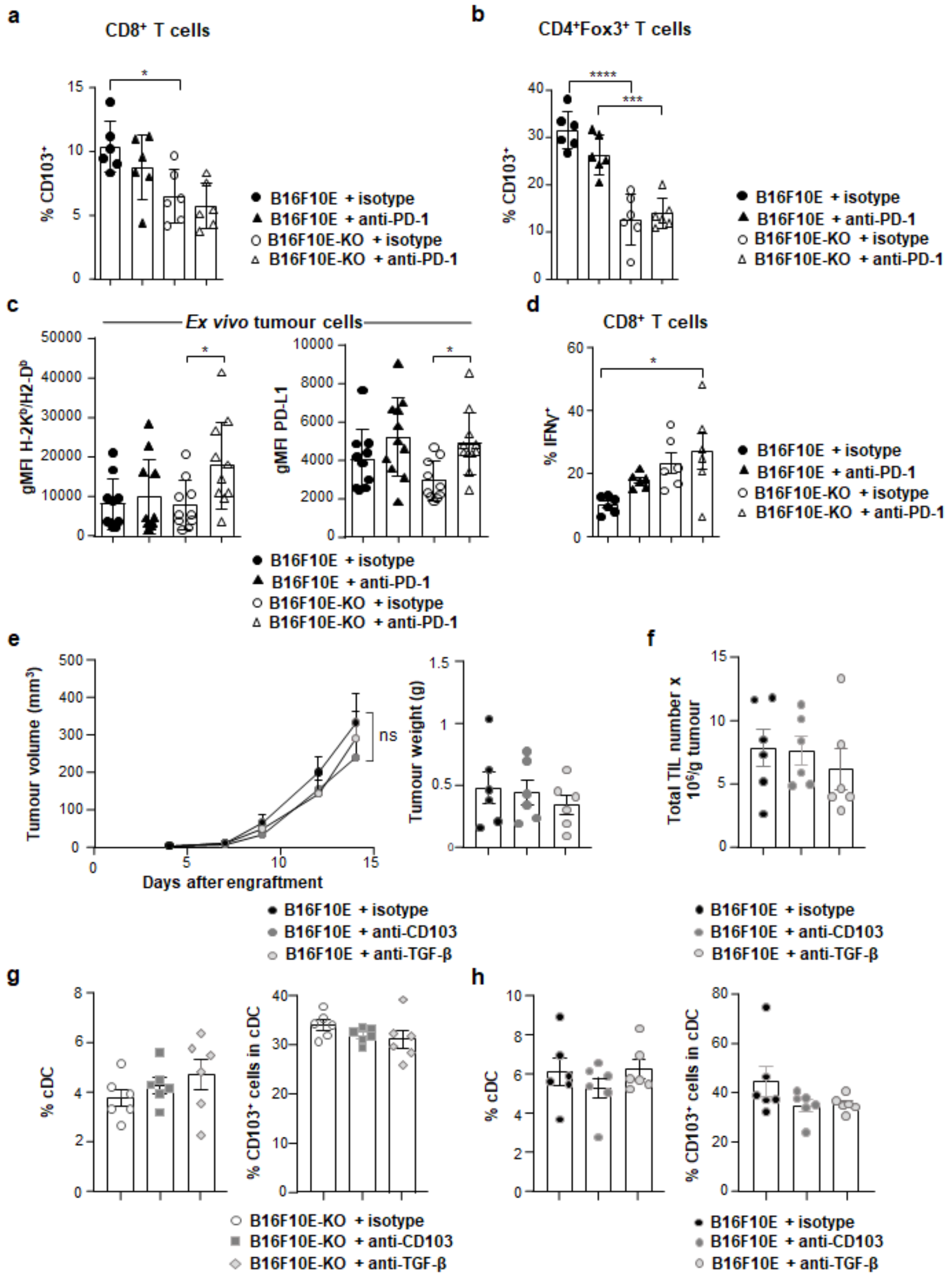

Supplementary Figure 7. Frequencies and phenotypic features of TIL in B16F10E and B16F10E-KO tumours. **a.** Percentages of $CD103^+$ cells among $CD8^+$ TIL from B16F10E and B16F10E-KO tumours (n=6), treated with anti-PD-1 mAb or isotype control, *p=0.024. Data are from one independent experiment out of three. **b.** Percentages of $CD103^+$ T cells among $CD4^+Foxp3^+$ TIL from B16F10E and B16F10E-KO tumours (n=6), treated with anti-PD-1 or isotype control, ***p=0.0004, ****p<0.0001. Data are from one independent experiment out of three. **c.** gMFI of H-$2K^b$/H-$2D^b$ expression on B16F10E (n=10) and B16F10E-KO cells (n=11) isolated *ex vivo* from mice treated with anti-PD-1 or isotype control, *p=0.020. Right, gMFI of PD-L1 expression on B16F10E (n=10) and B16F10E-KO cells (n=11), *p=0.045. **d.** Percentages of $IFN\gamma^+$ lymphocytes in $CD8^+$ T cells from B16F10E and B16F10E-KO tumours (n=6) treated with anti-PD-1 mAb or isotype control, *p=0.011. Data are from two independent experiments out of three. **e.** B16F10E tumour growth. Mice were engrafted with B16F10E cells and then treated i.t. with anti-TGF-β, anti-CD103 mAb or isotype control. Tumour volumes are means ± SEM of six mice/group, p=0.822. Right, weight of B16F10E tumours (n=6) recovered at day 14, p=0.656. **f.** Absolute number of TIL from B16F10E tumours (n=6) treated with anti-TGF-β, anti-CD103 or isotype control mAb, p=0.673. **g.** Percentages of conventional (c)DC in B16F10E-KO tumours (n=6) treated with isotype control, anti-CD103 or anti-TGF-β neutralizing mAb, p=0.357. Right, percentages of $CD103^+$ cDC in B16F10E-KO tumours (n=6), p=0.313. **h.** Percentages of cDC in B16F10E tumours (n=6), treated with isotype control, anti-CD103 or anti-TGF-β neutralizing mAb, p=0.503. Right, percentages of $CD103^+$ cDC in in B16F10E tumours (n=6), p=0.194. Each symbol represents an individual cell type. Horizontal lines are means ± SEM. Data were calculated with one-way ANOVA with Tukey's correction (a.- e. right, f.-h.), and two-way ANOVA (e. left). ns: non-significant. Source data are provided as a Source Data file.

Supplementary Table 1: Population description and immunohistochemistry data of the first anti-PD-(L)1 cohort (cohort 1)

Total number		Integrin α_V			
		Overall population	Integrin α_V (low)	Integrin α_V (high)	p-value
		N=106	N=89	N=17	
Histological subtype	Adenocarcinoma	62 (59%)	52 (58%)	10 (59%)	0.527
	Squamous	29 (27%)	23 (26%)	6 (35%)	
	NSCLC, other	15 (14%)	14 (16%)	1 (6%)	
Gender	Female	29 (27%)	23 (26%)	6 (35%)	0.423
	Male	77 (73%)	66 (74%)	11 (65%)	
Smoking status	Current-smoker	35 (33%)	31 (35%)	4 (24%)	0.663
	Former-smoker	61 (58%)	50 (56%)	11 (65%)	
	Non-smoker	10 (9%)	8 (9%)	2 (12%)	
Age (years)	<65	48 (45%)	39 (44%)	9 (53%)	0.489
	≥65	58 (55%)	50 (56%)	8 (47%)	
Advanced stage at diagnosis	III	12 (13%)	8 (9%)	4 (23%)	0.262
	IVA	27 (26%)	25 (28%)	2 (12%)	
	IVB	66 (63%)	55 (63%)	11 (65%)	
	NA	1	1	0	
Molecular status	*EGFR* mutation	5 (6%)	5 (8%)	0	1
	ALK fusion	2 (3%)	2 (3%)	0	1
	KRAS mutation	25 (32%)	20 (30%)	5 (42%)	0.438
	BRAF mutation	3 (4%)	3 (5%)	0	1
Previous therapies	Platinum-based chemotherapy	49 (46%)	39 (44%)	10 (59%)	0.361
	Other therapies	32 (30%)	27 (30%)	5 (29%)	
	No prior therapy	9 (8%)	9 (10%)	0	
	Chemoradiation	16 (15%)	14 (16%)	2 (12%)	
Number of metastatic sites	≤2	52 (49%)	43 (48%)	9 (53%)	0.727
	>2	54 (51%)	46 (52%)	8 (47%)	
Immunotherapy	Atezolizumab	13 (12%)	10 (11%)	3 (18%)	0.695
	Durvalumab	1 (1%)	1 (1%)	0	
	Nivolumab	69 (65%)	57 (64%)	12 (71%)	
	Pembrolizumab	23 (22%)	21 (24%)	2 (12%)	
Line of immunotherapy	≤2	65 (61%)	58 (65%)	7 (41%)	0.774
	>2	41 (39%)	31 (35%)	10 (59%)	
Density $CD8^+$ cells	High	62 (72%)	43 (77%)	11 (69%)	0.513
	Low	24 (28%)	13 (23%)	5 (31%)	
	Missing	20	33	1	
Performance status	0-1	89 (85%)	78 (88%)	11 (69%)	0.050
	≥2	16 (15%)	11 (12%)	5 (31%)	
	NA	1	0	1	
Response rate	CR	1 (1%)	1 (1%)	0	0.154
	PR	22 (22%)	19 (22%)	3 (19%)	
	SD	37 (36%)	33 (38%)	4 (25%)	
	PD	42 (41%)	33 (38%)	9 (56%)	
	NA	4	3	1	

Clinical characteristics of NSCLC patients treated with anti-PD-(L)1. CR: complete response; PR: partial response; SD: stable disease; PD: progression disease; NA: not available. All tests were two-sided, no adjustments were made for multiple comparisons.

Supplementary Table 2: Population description and immunohistochemistry data of the second anti-PD-(L)1 cohort (cohort 2)

Total number		Integrin α_V			
		Overall population	Integrin α_V (low)	Integrin α_V (high)	p-value
		N=51	N=42	N=9	
Histological subtype	Adenocarcinoma	39 (76%)	31 (74%)	8 (89%)	1
	Squamous	3 (6%)	3 (7%)	0	
	NSCLC, other	9 (18%)	8 (19%)	1 (11%)	
Gender	Female	23 (45%)	19 (45%)	4 (44%)	1
	Male	28 (55%)	23 (55%)	5 (56%)	
Smoking status	Current-smoker	21 (41%)	19 (45%)	2 (22%)	0.339
	Former-smoker	24 (47%)	18 (43%)	6 (67%)	
	Non-smoker	6 (12%)	5 (12%)	1 (11%)	
Age (years)	<65	27 (53%)	20 (47%)	7 (78%)	0.147
	≥ 65	24 (47%)	22 (52%)	2 (22%)	
Stage advance diagnosis	IIA	1 (2%)	1 (2%)	0	0.772
	IIB	1 (2%)	1 (2%)	0	
	IVA	11 (22%)	10 (24%)	1 (11%)	
	IVB	38 (74%)	30 (72%)	8 (89%)	
Molecular status	*EGFR* mutation	4 (8%)	3 (8%)	1 (11%)	1
	ALK fusion	2 (4%)	1 (3%)	1 (11%)	0.337
	KRAS mutation	18 (38%)	14 (36%)	4 (44%)	0.711
	BRAF mutation	2 (4%)	2 (5%)	0	1
Previous therapies	Platinum-based chemotherapy	29 (57%)	25 (60%)	4 (44%)	0.401
	Other therapies	11 (22%)	9 (21%)	2 (22%)	
	No prior therapy	4 (8%)	3 (7%)	1 (11%)	
	NA	4 (7%)	2 (5%)	2 (23%)	
	Chemoradiation	3 (6%)	3 (7%)	0	
Immunotherapy	Nivolumab	26 (51%)	22 (52%)	4 (44%)	0.726
	Pembrolizumab	25 (49%)	20 (48%)	5 (56%)	
Line of immunotherapy	≤2	38 (75%)	33 (79%)	5 (56%)	0.208
	>2	13 (25%)	9 (21%)	4 (44%)	
Performance status	0-1	29 (57%)	25 (60%)	4 (44%)	0.076
	≥2	22 (43%)	17 (40%)	5 (56%)	
Response rate	CR	2 (4%)	2 (8%)	0	0.343
	PR	10 (20%)	7 (17%)	3 (33%)	
	SD	10 (20%)	10 (24%)	0	
	PD	26 (51%)	21 (50%)	5 (56%)	
	NA	3 (5%)	2 (1%)	1 (11%)	

Clinical characteristics of NSCLC patients treated with anti-PD-(L)1. CR: complete response; PR: partial response; SD: stable disease; PD: progression disease; NA: not available. All tests were two-sided, no adjustments were made for multiple comparisons.

Supplementary Table 3: Multivariable analysis for progression free survival (PFS) performed on the pooled cohort

	HR (95%CI)	p-value
Age > 65 years	1.21 (0.84-1.75)	0.31
Smokers	0.40 (0.23-0.70)	0.001
Line of immunotherapy > 2	1.23 (0.84-1.81)	0.29
Performance status ≥2	1.61 (1.07-2.43)	0.02
α_V^{high}	1.60 (0.98-2.62)	0.06

Analyses were performed on cohort 1 plus cohort 2 (n=157). HR: hazard ratio; CI: confidence interval. All tests were two-sided, no adjustments were made for multiple comparisons.

Supplementary Table 4: Integrin α_V, β_6 and β_8 protein expression in NSCLC cell lines

	α_v	β_6	β_8
NSCLC cell lines	% (gMFI)	% (gMFI)	% (gMFI)
LCC			
IGR-Heu	1%	0%	0%
IGR-B2	100% (76651)	100% (65366)	21% (30319)
H1155	4%	0%	0%
H460	3%	3%	0%
ADC			
ADC-CocoT7	60% (67878)	0%	1%
IGR-Pub	95% (69211)	0%	0%
ADC-Tor	61% (91263)	0%	0%
ADC-Let	39% (97887)	0%	0%
A549	90% (84607)	5%	0%
H1355	23% (32197)	0%	1%
SCC			
Lud-Lu	8%	0%	0%
SK-MES	96% (55538)	0%	0%
SCLC			
DMS-53	19% (26974)	49% (32754)	0%

Percentages and gMFI are included. 16HBE human bronchial epithelial cells were used as a control.

Supplementary Table 5: Expression of *ITGB1*, *ITGB3* and *ITGB*5, and *TGFB1* mRNA in NSCLC cell lines

	ITGB1	*ITGB3*	*ITGB5*	*TGFB1*
NSCLC cell lines				
LCC				
IGR-B2	1.1	6.9	0.0	1.8
IGR-Heu	1.2	0	0.8	0.3
H1155	0.2	7.7	0.1	2.4
H460	0.6	0	0.3	0.5
ADC				
ADC-CocoT7	2.9	12.4	3.6	1.3
IGR-Pub	3.8	2.7	1.6	1.1
ADC-Tor	7.7	4.0	2.4	7.2
ADC-Let	1.3	8.0	0.8	1
A549	3.1	35.0	1.8	2
H1355	1.3	2.0	0.8	0.4
SCC				
Lud-Lu	0.8	0	0.3	1
SK-MES	3.3	0.6	1.0	0.5
SCLC				
DMS-53	0.2	0.4	0.2	0.7

Expression was normalized to *18S* expression. 16HBE human bronchial epithelial cells were used as a control. High expression is in bold. Data are presented as fold changes.

Supplementary Table 6: Primer pairs used for qRT-PCR

Human primer pairs

	5’ Forward primer 3’	3’ Reverse primer 5’
ITGAV	GCACCAGCAGTCAGAGATG	TGAACAACTGGCCCAACATC
ITGB1	GGGAGCCACAGACATTTACATT	CCGAAGTAATCCTCCTCATTTCA
ITGB3	AACTGTGCCCCAGAATCCAT	AATCCTCTGGGGACTGACTTGA
ITGB5	TGATCTGAGGGCAAACCTTGT	GCAATCTCCTGTGGTGTCATCT
ITGB6	ATCTGGAGTTGGCGAAAGG	CTTTGAGGCGCAATCTGAAC
ITGB8	CCACCCCGAAAGGATTCATAAT	TGGCGTCAAAACCTCCTTCT
TGFB1	GCAACAATTCCTGGCGATACCT	GCTAAGGCGAAAGCCCTCAAT
18S	CGGACAGGATTGACAGATTG	CAAATCGCTCCACCAACT

Mouse primer pairs

	5’ Forward primer 3’	3’ Reverse primer 5’
itgb1	ATGCCAAATCTTGCGGAGAAT	TTTGCTGCGATTGGTGACATT
itgb3	GTCACATTGGCACCGACAACC	CCACACTCAAAAGTCCCGTTC
itgb5	GATGCGGTCCTCCAGGCTGC	CACTGGCCATCGTGGGGCTG
itgb6	ACTACCCTTCATTGAAAACCCTG	TGAGGAGCAATCTGAACAATGTC
itgb8	TCCTCTGAAGAAATACCCCGTG	TGGCGAGACAGTTTTATCCACA
18S	CGGACAGGATTGACAGATTG	CAAATCGCTCCACCAACT
