# Supplementary material for: Integrin-αV-mediated activation of TGF-β regulates anti-tumour CD8 T cell immunity and response to PD-1 blockade
Source: Nat Commun. 2021 Sep 1;12:5209. doi: 10.1038/s41467-021-25322-y (PMC8410945; doi:10.1038/s41467-021-25322-y)
Supplement: Supplementary file 2 — Reporting Summary [file 41467_2021_25322_MOESM2_ESM.pdf]

nature research

Corresponding author(s): Fathia Mami-Chouaib

Last updated by author(s): Jun 10, 2021

# Reporting Summary

Nature Research wishes to improve the reproducibility of the work that we publish. This form provides structure for consistency and transparency in reporting. For further information on Nature Research policies, see our Editorial Policies and the Editorial Policy Checklist.

## Statistics

For all statistical analyses, confirm that the following items are present in the figure legend, table legend, main text, or Methods section.

| n/a | Confirmed | |
|---|---|---|
| ☐ | ☒ | The exact sample size (*n*) for each experimental group/condition, given as a discrete number and unit of measurement |
| ☐ | ☒ | A statement on whether measurements were taken from distinct samples or whether the same sample was measured repeatedly |
| ☐ | ☒ | The statistical test(s) used AND whether they are one- or two-sided<br>*Only common tests should be described solely by name; describe more complex techniques in the Methods section.* |
| ☐ | ☒ | A description of all covariates tested |
| ☐ | ☒ | A description of any assumptions or corrections, such as tests of normality and adjustment for multiple comparisons |
| ☐ | ☒ | A full description of the statistical parameters including central tendency (e.g. means) or other basic estimates (e.g. regression coefficient) AND variation (e.g. standard deviation) or associated estimates of uncertainty (e.g. confidence intervals) |
| ☐ | ☒ | For null hypothesis testing, the test statistic (e.g. *F*, *t*, *r*) with confidence intervals, effect sizes, degrees of freedom and *P* value noted<br>*Give P values as exact values whenever suitable.* |
| ☒ | ☐ | For Bayesian analysis, information on the choice of priors and Markov chain Monte Carlo settings |
| ☒ | ☐ | For hierarchical and complex designs, identification of the appropriate level for tests and full reporting of outcomes |
| ☒ | ☐ | Estimates of effect sizes (e.g. Cohen's *d*, Pearson's *r*), indicating how they were calculated |

*Our web collection on statistics for biologists contains articles on many of the points above.*

## Software and code

Policy information about availability of computer code

| | |
|---|---|
| Data collection | Multispectral fluorescent images were captured using the Vectra 3 microscope (PerkinElmer). Flow cytometry data were collected on BD Fortessa instruments with FACSDiva software V10 (BD). Relative luciferase activity data were collected on In Vivo Imaging System IVIS (PerkinElmer). qRT-PCR data were collected with rtqPCR step-one Plus (Applied). Proximity ligation assay (PLA) data were collected with confocal microscope SP8 (Leica). |
| Data analysis | Image analysis of multispectral fluorescent images was performed using InForm software (PerkinElmer). Using http://kmplot.com, analysis of gene-expression TCGA datasets derived from NSCLC patients was performed. Gene-expression data were automatically computed to generate Kaplan-Meier from TCGA datasets. Flow cytometry data were analyzed on FlowJo v10 (TreeStar) and statistical analysis performed on Prism v8 (GraphPad). Relative luciferase activity data were calculted using ROI measurements by IVIS (PerkinElmer). PLA experiments were analyzed with Icy software (Gustave Roussy). The proliferation index was calculated every other day by flow cytometric analysis using ModFit LT™ program v5.0. Regarding patients cohorts, the best cut-point for total CD8+, CD8+CD103+ and CD8+CD103neg TIL was assessed using the log-rank maximization method. Survival analyses were performed using the Kaplan-Meier method and the log-rank test. All p-values inferior to 0.05 were considered statistically significant. A Cox proportional hazards regression model was used to evaluate independent prognostic factors for OS and PFS. Variables included in the final multivariate model were selected according to their clinical relevance and statistical significance in univariate analysis (p-value cut-off = 0.10). The proportional hazard hypothesis was verified with the Schoenfeld residual method. Predictive factors of disease control were tested with logistic regression in univariate and multivariate analyses. The alpha level was 5%. Statistical analyses were performed with RStudio v1.1.463 (free software environment for statistical computing and graphics). |

For manuscripts utilizing custom algorithms or software that are central to the research but not yet described in published literature, software must be made available to editors and reviewers. We strongly encourage code deposition in a community repository (e.g. GitHub). See the Nature Research guidelines for submitting code & software for further information.

## Data

Policy information about availability of data

All manuscripts must include a data availability statement. This statement should provide the following information, where applicable:

- Accession codes, unique identifiers, or web links for publicly available datasets
- A list of figures that have associated raw data
- A description of any restrictions on data availability

The authors state that all data generated during this study are included in the article and its Supplementary Information file and are available from the corresponding author upon request. Source data are provided with this paper. The use of publicly available data from lung cancer cohorts were consulted on the website https://kmplot.com/analysis/index.php?p=service&cancer=lung , under the specific data product name: KM Plotter – Lung Cancer.

# Field-specific reporting

Please select the one below that is the best fit for your research. If you are not sure, read the appropriate sections before making your selection.

☒ Life sciences ☐ Behavioural & social sciences ☐ Ecological, evolutionary & environmental sciences

For a reference copy of the document with all sections, see nature.com/documents/nr-reporting-summary-flat.pdf

# Life sciences study design

All studies must disclose on these points even when the disclosure is negative.

| | |
|---|---|
| Sample size | No sample size calculation was performed for in vitro studies. Every in vitro experiment was performed independently at least 3 times for subsequent statistical analysis. Animal sample size were determined using previous studies and guided by the 3R principle. Human samples sizes were driven by the availability of biopsies. |
| Data exclusions | No data were excluded from the analysis. |
| Replication | We established two cohorts of NSCLC patients treated with anti-PD(L)1 immunotherapy. Regarding the number of patients, the first cohort included 106 patients, whereas the second cohort included 51 patients. The attempt to replicate the same number of patients in both cohorts was not achieved due to the long inclusion period of patients. Moreover, the results were reliably reproduced for in vitro and in vivo experiments. This included independant replication: experiments presented in this study were performed using at least 2 biological replicates, except the experiment with Nude mice or WT C57Bl/6 mice engrafted with B16F10E or B16F10E-KO and treated in parallel with anti-PD-1, a-TGFβ or a-CD103, which were performed once. |
| Randomization | Human material was obtained from random healthy donors independently of sex, age and any other characteristics. NSCLC patients were selected based on the eligibility for the operation after informed written consent and based on the disponibility of the biological material after pathology examinations. Mice were randomized after tumour inoculation to obtain homogeneous groups. |
| Blinding | Blinding was used during measurement of tumours in different treatment groups, and also during the analysis where each mouse was sorted by their numbers and not by the experimental group. The assessment of αV staining was performed by an expert thoracic pathologist blinded from clinical data. For assessment of αV staining experiments, the investigators were blinded to group allocation during data collection. For in vitro experiments, the blinding was not relevant because the analysis of flow cytometry data was performed with fixed gates for each group. |

# Reporting for specific materials, systems and methods

We require information from authors about some types of materials, experimental systems and methods used in many studies. Here, indicate whether each material, system or method listed is relevant to your study. If you are not sure if a list item applies to your research, read the appropriate section before selecting a response.

## Materials & experimental systems

| n/a | Involved in the study | |
|---|---|---|
| ☐ | ☒ | Antibodies |
| ☐ | ☒ | Eukaryotic cell lines |
| ☒ | ☐ | Palaeontology and archaeology |
| ☐ | ☒ | Animals and other organisms |
| ☐ | ☒ | Human research participants |
| ☒ | ☐ | Clinical data |
| ☒ | ☐ | Dual use research of concern |

## Methods

| n/a | Involved in the study | |
|---|---|---|
| ☒ | ☐ | ChIP-seq |
| ☐ | ☒ | Flow cytometry |
| ☒ | ☐ | MRI-based neuroimaging |

## Antibodies

**Antibodies used**

For IHC:
anti- αV integrin αV (clone EPR16800, Abcam ab179475, 1/1000), -CD8 (clone SP16, Spring Bioscience M3160, 1/200), aanti-CD103 (clone EPR4166-2, Abcam ab129202, 1/200) and anti-cytokeratin (clones AE1/AE3, Dako GA05361-2, 1/100) mAb

For flow cytometry:
Human: anti-αV (clone NKI-M9, BioLegend 327908, 1/100), -β6 (clone 437211, R&D Systems MAB4155, 1/100), -β8 (clone 416922, R&D Systems MAB4775, 1/100), -E-cadherin (clone 67A4, BioLegend 324106, 1/200) and -EpCAM (clone 9C4, BioLegend 324207, 1/200), -CD3 (clone UCHT1, BioLegend 300463, 1/200), -CD8α (clone RPA-T8, BioLegend 301012, 1/200), -CD4 (clone RPA-T4, BioLegend 300506, 1/200), -CD103 (clone Ber-ACT8, BioLegend 350204, 1/100), KLRG1 (clone 13F12F2, ThermoFicher 12-9488-42, 1/100) mAb
Mouse: anti-CD51 (integrin αV, clone RMV-7, BioLegend 104106, 1/200), E-cadherin (clone DECMA-1, BioLegend 147307, 1/200), H2-Kb/H2-Db (clone 28-8-6, BioLegend 114605, 1/200), PD-L1 (clone 10F.9G2, BioLegend 124313, 1/100), integrin β5 (clone KN52, Thermo Fischer Scientific 11-0497-41, 1/50), integrin β3 (clone 909114, R&D systems FAB8557A, 1/100), CD3 (clone 17A2, BioLegend 100241, 1/200), CD4 (clone RM4-5, BioLegend 100536, 1/200), FoxP3 (clone FJK-16S, Thermo Ficher 35-5773-80, 1/100), CD8α (clone REA601, Miltenyi 130-109-252, 1/20), CD62L (clone MEL-14, BioLegend 104405, 1/200), CD44 (clone IM7, BioLegend 103030, 1/100), PD-1 (clone 29F.1A12, BioLegend 135223, 1/100), CD69 (clone H1.2F3, BioLegend 104512, 1/100), CD103 (clone 2E7, BioLegend 121418, 1/200), KLRG1 (clone 2F1, BioLegend 138407, 1/100), Ki-67 (clone REA183, Miltenyi 130-120-556, 1/11), IFN-γ (clone XMG1.2 BioLegend 505825, 1/50), granzyme B (clone GB11, BioLegend 515403, 1/50), pSmad2/3 (clone 072-670, BD 562586, 1/20), MHC-II (clone REA528, Miltenyi 130-108-004, 1/20), CD11c (clone N418, BioLegend 117321, 1/200). Staining of MMP14 was performed with specific mAb (clone EP1264Y, Abcam ab51074, 1/200) followed by secondary antibody staining (goat anti-rabbit IgG Abcam ab150077, 1/2000)
For PBMC stimulation:
anti-CD3 (clone OKT3, BioLegend 317301, 1/1000), anti-TGF-β (clone 1D11.16.8, compagnie BioXcell BE0057, 10µg/ml) mAb
For PLA:
anti-αV (clone EPR16800, Abcam ab179475, 1/100) and anti-LAP-TGF-β (clone TW7-16B4, BioLegend 141402, 1/100) mAb
For functional assay and in vivo experiments (mouse):
anti-CD8α (clone 2.43, Bio-X-Cell BE0061, 100 µg/mouse), anti-PD-1 (clone RMP1-14, Bio-X-Cell BE0146, 200 µg/mouse), anti-CD103 (clone M290, Bio-X-Cell BE0026, 50 µg/mouse), anti-TGFβ (clone 1D11.16.8, Bio-X-Cell BE0057, 25 µg/mouse); mAb and isotype controls (IgG2b, clone LTF-2 Bio-X-Cell BE0090; IgG2a: clone 2A3, Bio-X-Cell BE0089, IgG1: clone MOPC-21, Bio-X-Cell BE0083) mAb
ELISA:
Antibodies in TGF-β DuoSet ELISA kits (DY240 and DY1679, ThermoFischer Scientific).

**Validation**

All commercial antibodies were used (assay and species) according to the manufacturer's instructions. Validation of flow cytometry antibodies was performed by the titration to determine the optimal concentration through the series of the dilutions: 1/400, 1/200, 1/100, 1/50, 1/25, 1/11. Optimal concentration was defined by calculating the stain index.
Human anti-β6 (clone 437211, R&D Systems MAB4155, 1/100) was conjugated to PE/Cy7 fluorophore with Lighting link strategy by Innova Biosciences (762-0030), following the manufacturer's instructions (https://www.abcam.com/pecy7reg-conjugation-kit-lightning-linkreg-ab102903.html). The succesfull conjugation was validated by comparing the flow cytometry plots of integrin β6 expression, using unconjugated human anti-β6 (clone 437211, R&D Systems MAB4155, 1/100), follwed by secondary PE-Cy7 antibody staining.

## Eukaryotic cell lines

Policy information about cell lines

**Cell line source(s)**

Human cell line:
The IGR-B2 cell line and the autologous B90 CTL clone were derived from patient Bla large cell carcinoma (Le Floc'h A, et al. Alpha E beta 7 integrin interaction with E-cadherin promotes antitumor CTL activity by triggering lytic granule polarization and exocytosis. J Exp Med 204, 559-570 (2007)). The allogeneic NSCLC cell lines IGR-Pub, IGR-Heu, ADC-Coco, ADC-Tor and ADC-Let were derived from tumour specimens in one of our laboratories. H1355 (adenocarcinoma), H460 and H1155 (large cell carcinoma) were a generous gift from Dr. S. Rogers (Brigham and Women's Hospital, Boston, MA), and A549 (adenocarcinoma), SK-Mes, Ludlu (squamous cell carcinoma, SCC) and DMS53 (small-cell lung carcinoma, SCLC) were purchased from the European Collection of Cell Cultures (Le Floc'h A, et al. Alpha E beta 7 integrin interaction with E-cadherin promotes antitumor CTL activity by triggering lytic granule polarization and exocytosis. J Exp Med 204, 559-570 (2007). 16HBE14o- (16HBE) (Merck, SCC150).

Mouse cell line:
The B16F10 melanoma cell line (H-2b) was purchased from the American Type Culture Collection (ATCC, CRL-6475™ ).

Mink lung epithelial cell line Mu.1LV was a gift from Céline Prunier, Centre de Recherche Saint Antoine, Paris.

**Authentication**

We regularly authenticate our NSCLC cell lines by testing recognition by autologous CTL clones and HLA-A2 expression when applicable. Purchased B16F10 cell line was visualy authenticated. The reporter mink lung epithelial cell line Mu.1LV was not authenticated.

**Mycoplasma contamination**

All the cell lines are mycoplasma-free and were regularly tested for mycoplasma contamination.

**Commonly misidentified lines**
(See ICLAC register)

No commonly misidentified cell lines were used in the study.

## Animals and other organisms

Policy information about studies involving animals; ARRIVE guidelines recommended for reporting animal research

| | |
|---|---|
| Laboratory animals | Female C57BL/6J mice (7-9 weeks old) were purchased from Envigo. The female athymic nude Crl:NU(NCr)-Foxn1nu mice (6 weeks) were inbred in-house. Mice were housed in Super Mouse 750TM ventilated cages. Cages contained 1/8" corn cob bedding. Lights were on a 12h ON/OFF cycle, room temperature was set to 22°C with the variance of +/- 2°C and ambient humidity conditions. A standard food diet (Envigo) and water (purified by reverse osmosis) were provided ad libitum. |
| Wild animals | Study did not involve wild animals. |
| Field-collected samples | Study did not involve samples collected in the field. |
| Ethics oversight | All animals were housed at Gustave Roussy's animal facility and treated in accordance with guidelines established by the institutional animal committee (CEEA n°026: 2018-056-16280), after receiving the legal approval from French Minister of Higher Education, Research and Innovation under the procedure number APAFIS#16281-2018072515064652v2. |

Note that full information on the approval of the study protocol must also be provided in the manuscript.

## Human research participants

Policy information about studies involving human research participants

| | |
|---|---|
| Population characteristics | Healthy donor blood samples were collected from the French blood bank (Etablissement français du Sang (EFS); agreement number N°12/EFS/079). Fresh NSCLC tumours were obtained from the Centre chirurgical Marie Lannelongue. FFPE tumour samples from NSCLC patients were obtained from patients treated with anti-PD(L)1 immunotherapy in a variety of settings in Gustave Roussy between 2012 and 2020. In the first imunotherapy-treated cohort, 55% of patients were over 65y old, 73% were male, 32% had KRAS mutated NSCLC, 46% received platinum-based chemotherapy, 15% chemoradiation, 30% other therapies and 8% had no prior therapy before received anti-PD-(L)1 immunotherapy, and 59% had adenocarcinoma. In the second imunotherapy-treated cohort, 47% of patients were over 65y old, 55% were male, 38% had KRAS mutated NSCLC, 57% received platinum-based chemotherapy, 6% chemoradiation, 22% other therapies and 8% had no prior therapy before received anti-PD-(L)1 immunotherapy, and 76% had adenocarcinoma. The treatment-naïve cohort of NSCLC patients has been previously described (Djenidi F, et al. CD8+CD103+ tumor-infiltrating lymphocytes are tumor-specific tissue-resident memory T cells and a prognostic factor for survival in lung cancer patients. J Immunol 194, 3475-3486 (2015). |
| Recruitment | Human blood material was obtained from random healthy donors from the French blood bank. Human samples from NSCLC patients were obtained from the Centre chirurgical Marie Lannelongue after informed written consent. We randomly selected samples from healthy donors, or patients. All the samples were anonymized. No other self-selection biases are present. |
| Ethics oversight | Etablissement français du Sang (EFS); agreement number N°12/EFS/079 for blood samples from healthy donors. For NSCLC samples, the study was approved by the Institutional Review Board of Gustave Roussy (Commission Scientifique des Essais Thérapeutiques [CSET]) and informed consent from patients was obtained. |

Note that full information on the approval of the study protocol must also be provided in the manuscript.

## Flow Cytometry

### Plots

Confirm that:

☒ The axis labels state the marker and fluorochrome used (e.g. CD4-FITC).

☒ The axis scales are clearly visible. Include numbers along axes only for bottom left plot of group (a 'group' is an analysis of identical markers).

☒ All plots are contour plots with outliers or pseudocolor plots.

☒ A numerical value for number of cells or percentage (with statistics) is provided.

### Methodology

| | |
|---|---|
| Sample preparation | For human experiments:<br>For freshly isolated TIL and tumour cells, human lung tumours were dissociated mechanically and enzymatically using a Tumor Dissociation Kit (Miltenyi, 130-095-929). Mononuclear cells were then isolated by a Ficoll-Hypaque gradient. Tumour cells were isolated by magnetic separation using Tumor Cell Isolation Kit (Miltenyi, 130-108-339). For PBL, mononuclear cells were then isolated by a Ficoll-Hypaque gradient.<br>Cell surface and intracellular staining of human cells was performed on single-cell suspensions. Dead cells were excluded using the Live/Dead Fixable Blue Dead Cell Stain Kit (Invitrogen 186684). For intracellular staining, cells were fixed, permeabilized with the FoxP3 staining buffer set according to the manufacturer's instructions (eBioscience 00-5523-00).<br>For mouse experiments:<br>Tumours were harvested at day 14-17 and digested for 30 min at 37°C according to the Tumor Dissociation kit protocol (130-096-730, Miltenyi). Tumours were crushed on 100 µm cell strainers and washed twice with PBS 2% FCS. Single cell suspensions were enriched for CD45+ or CD8+ cells using anti-CD45 or anti-CD8 microbeads (130-052-301 or 130-117-044, |

| | |
|---|---|
| | Miltenyi), and then purified using the POSSEL2 program on MultiMACS. The positive fraction was recovered for TIL analysis and the negative fraction for tumour cell analysis by flow cytometry. Cell surface and intracellular staining of mouse cells was performed on single-cell suspensions. Dead cells were excluded using the Live/Dead Fixable Blue Dead Cell Stain Kit (Invitrogen 186684). For intracellular staining, cells were fixed/permeabilized with the Foxp3 Staining Buffer Set according to the manufacturer's instructions (eBioscience 00-5523-00). |
| Instrument | BD Fortessa and MultiMACS system (Miltenyi Biotec). |
| Software | FlowJo v10 (TreeStar). |
| Cell population abundance | Purity of cell population isolated by FACS or MultiMACS was typically >90%.<br>CD8 T cells from tumours represent at least >5% of the CD45+ cells isolated (mouse experiment). |
| Gating strategy | To analyse cells a preliminary FSC/SSC gate was utilized to gate on the morphology of lymphocytes or tumoural cells. Subsequently a singlets gate (FSC-H vs FSC-A) was used to exclude the doublets followed by the viability gate (Live/Dead UV vs FSC-A) to exclude dead cells. From this population, relevant gating strategies are described. Please refer to Supplementary Figures 2c, 3a and 5c. |

☒ Tick this box to confirm that a figure exemplifying the gating strategy is provided in the Supplementary Information.
